# Integration of enzyme activities into metabolic flux distributions by elementary mode analysis

**DOI:** 10.1186/1752-0509-1-31

**Published:** 2007-07-18

**Authors:** Hiroyuki Kurata, Quanyu Zhao, Ryuichi Okuda, Kazuyuki Shimizu

**Affiliations:** 1 Department of Bioscience and Bioinformatics, Kyushu Institute of Technology, 680-4 Kawazu, Iizuka, Fukuoka, 820-8502, Japan

## Abstract

**Background:**

In systems biology, network-based pathway analysis facilitates understanding or designing metabolic systems and enables prediction of metabolic flux distributions. Network-based flux analysis requires considering not only pathway architectures but also the proteome or transcriptome to predict flux distributions, because recombinant microbes significantly change the distribution of gene expressions. The current problem is how to integrate such heterogeneous data to build a network-based model.

**Results:**

To link enzyme activity data to flux distributions of metabolic networks, we have proposed Enzyme Control Flux (ECF), a novel model that integrates enzyme activity into elementary mode analysis (EMA). ECF presents the power-law formula describing how changes in enzyme activities between wild-type and a mutant are related to changes in the elementary mode coefficients (EMCs). To validate the feasibility of ECF, we integrated enzyme activity data into the EMCs of *Escherichia coli *and *Bacillus subtilis *wild-type. The ECF model effectively uses an enzyme activity profile to estimate the flux distribution of the mutants and the increase in the number of incorporated enzyme activities decreases the model error of ECF.

**Conclusion:**

The ECF model is a non-mechanistic and static model to link an enzyme activity profile to a metabolic flux distribution by introducing the power-law formula into EMA, suggesting that the change in an enzyme profile rather reflects the change in the flux distribution. The ECF model is highly applicable to the central metabolism in knockout mutants of *E. coli *and *B. subtilis*.

## Background

In systems biology, a mathematical approach is required to integrate heterogeneous data, such as transcriptome, proteome, metabolome, and fluxome, to build comprehensive metabolic models. Mathematical models are consistently improved or modified by accommodating new experimental data with the current models, thereby enhancing the validation of metabolic networks or the prediction of their dynamic behaviors[1,2].

Network-based pathway analysis becomes a core method for constructing a mathematical model that predicts the flux distribution for large-scale metabolic networks. Network-based analysis generally employs a constraint-based modelling approach [3], e.g., Flux Balance Analysis (FBA) that uses a stoichiometric matrix and objective function that define a network's allowable solution space. The target flux capacity is provided by optimizing specific objective functions such as cell growth, energy, or metabolite synthesis maximization[3,4].

Recent network-based metabolic pathway analysis has focused on two approaches, elementary modes (EMs) [5] and extreme pathways[6]. Both consist of a convex set of vectors used to characterize all steady-state flux distributions of a biochemical network. The elementary mode is the minimal set of enzymes that can operate at steady-state, with all the irreversible reactions operating properly. Elementary mode or extreme pathway analysis enables an understanding of a large-scale network, predicting optimal or suboptimal metabolic fluxes under constrained conditions.

Since some genetic or environmental perturbations often cause a significant change in gene expression, a network-based flux analysis requires considering not only pathway architectures but also the proteins or mRNA transcripts to predict a flux distribution. However, only a few methods have been developed that connect such heterogeneous data to network-based analyses[7,8], e.g., the constraints from absent model genes with Boolean logic were employed. A current problem is to explore the relationship between an enzyme activity profile and a metabolic flux distribution, or how to mathematically integrate such heterogeneous data to build a network-based model.

To elucidate theoretical relationships between transcriptome or enzyme activities and flux, the control-effective flux (CEF) method has previously been proposed to predict gene expression (enzyme) levels through elementary mode analysis[9], but CEF does not estimate metabolic flux distributions from transcriptome or enzyme activities data. In our limited knowledge, there are few efficient mathematical models that use an enzyme activity profile to estimate a metabolic flux distribution. The mathematical connections between enzymes and flux distributions are a very important task for a network-based model.

To link metabolic enzyme data to flux distributions, we propose Enzyme Control Flux (ECF), a non-mechanistic and static method that integrates enzyme activities into EMA. The ECF model presents a novel mathematical formula describing how the change in enzyme activities between wild-type and a mutant are related to that in the EM coefficients (EMCs). To validate the feasibility of ECF, we integrated enzyme activity data into the EMCs of the central metabolism of *E. coli *and *B. subtilis *wild-type to simulate the metabolic flux distributions of the mutants.

## Results and discussion

**Figure 1:**
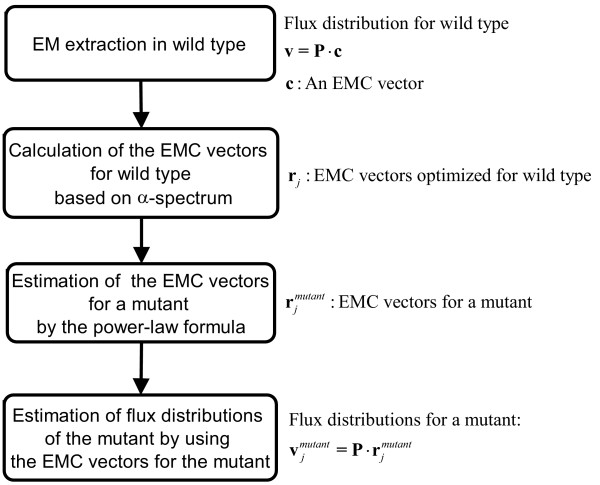
A flow chart for the ECF-based estimation of a flux distribution of a mutant.

The algorithm of ECF that links an enzyme activity profile to a metabolic flux distribution is presented as shown in Figure 1. The mathematical procedure of ECF is intelligibly illustrated (see Additional file 1). The power law formula uses the change in an enzyme activity profile between wild-type and a mutant to calculate the EMCs of the mutant, thereby simulating the flux distribution of the mutant. To guarantee the generality and applicability of ECF, we simulate the flux distributions of *E. coli *and *B. subtilis *mutants by integrating the change in an enzyme activity profile into the EMCs of their wild-type.

### ECF algorithm

#### Elementary mode analysis

Biological networks can be represented by a stoichiometric matrix (**S**). The rows of **S **correspond to metabolites in a reaction network. The columns of **S **correspond to the reactions in a network, with elements corresponding to stoichiometric coefficients of the associated reactions. At steady-state, mass balance provides the flux-balance equations:

**S·v = 0**,

where **v **= (*v*_1_, *v*_2_, ..., *v*_*n*_)^*t *^is the vector whose elements correspond to fluxes through the associated reactions in **S**. The set of all possible solutions can be described by a set of basis vectors. The elementary mode (EM) is the minimal set of enzymes that can operate at steady-state with all the irreversible reactions operating properly[5]. The elementary mode matrix (**P**) is uniquely determined from the stoichiometric matrix and the flux vector is provided by:

**v = P·c**,

where **c **= (*λ*_1_, *λ*_2_, ..., *λ*_*m*_)^*t *^is the EMC vector, *n *is the number of reactions, and *m *is the number of EMs. The ingredients of these vectors and matrix are displayed as:

(v1v2...vn)=(e11e12.....e1me21e22.....e2m........................en1en2.....enm)(λ1λ2.....λm).
 MathType@MTEF@5@5@+=feaafiart1ev1aqatCvAUfKttLearuWrP9MDH5MBPbIqV92AaeXatLxBI9gBaebbnrfifHhDYfgasaacH8akY=wiFfYdH8Gipec8Eeeu0xXdbba9frFj0=OqFfea0dXdd9vqai=hGuQ8kuc9pgc9s8qqaq=dirpe0xb9q8qiLsFr0=vr0=vr0dc8meaabaqaciaacaGaaeqabaqabeGadaaakeaadaqadaqaauaabeqageaaaaqaaiabdAha2naaBaaaleaacqaIXaqmaeqaaaGcbaGaemODay3aaSbaaSqaaiabikdaYaqabaaakeaacqGGUaGlaeaacqGGUaGlaeaacqGGUaGlaeaacqWG2bGDdaWgaaWcbaGaemOBa4gabeaaaaaakiaawIcacaGLPaaacqGH9aqpdaqadaqaauaabeqagGaaaaaaaeaacqWGLbqzdaWgaaWcbaGaeGymaeJaeGymaedabeaaaOqaaiabdwgaLnaaBaaaleaacqaIXaqmcqaIYaGmaeqaaaGcbaGaeiOla4cabaGaeiOla4cabaGaeiOla4cabaGaeiOla4cabaGaeiOla4cabaGaemyzau2aaSbaaSqaaiabigdaXiabd2gaTbqabaaakeaacqWGLbqzdaWgaaWcbaGaeGOmaiJaeGymaedabeaaaOqaaiabdwgaLnaaBaaaleaacqaIYaGmcqaIYaGmaeqaaaGcbaGaeiOla4cabaGaeiOla4cabaGaeiOla4cabaGaeiOla4cabaGaeiOla4cabaGaemyzau2aaSbaaSqaaiabikdaYiabd2gaTbqabaaakeaacqGGUaGlaeaacqGGUaGlaeaacqGGUaGlaeaacqGGUaGlaeaacqGGUaGlaeaacqGGUaGlaeaacqGGUaGlaeaacqGGUaGlaeaacqGGUaGlaeaacqGGUaGlaeaacqGGUaGlaeaacqGGUaGlaeaacqGGUaGlaeaacqGGUaGlaeaacqGGUaGlaeaacqGGUaGlaeaacqGGUaGlaeaacqGGUaGlaeaacqGGUaGlaeaacqGGUaGlaeaacqGGUaGlaeaacqGGUaGlaeaacqGGUaGlaeaacqGGUaGlaeaacqWGLbqzdaWgaaWcbaGaemOBa4MaeGymaedabeaaaOqaaiabdwgaLnaaBaaaleaacqWGUbGBcqaIYaGmaeqaaaGcbaGaeiOla4cabaGaeiOla4cabaGaeiOla4cabaGaeiOla4cabaGaeiOla4cabaGaemyzau2aaSbaaSqaaiabd6gaUjabd2gaTbqabaaaaaGccaGLOaGaayzkaaWaaeWaaeaafaqabeacbaaaaaqaaGGaciab=T7aSnaaBaaaleaacqaIXaqmaeqaaaGcbaGae83UdW2aaSbaaSqaaiabikdaYaqabaaakeaacqGGUaGlaeaacqGGUaGlaeaacqGGUaGlaeaacqGGUaGlaeaacqGGUaGlaeaacqWF7oaBdaWgaaWcbaGaemyBa0gabeaaaaaakiaawIcacaGLPaaacqGGUaGlaaa@903F@

The *i*-th column for the **P **matrix is the *i*-th EM vector: **e**_i _= (*e*_1*i*_, *e*_2*i*_, ..., *e*_*ni*_)^*t*^. The flux distribution can be also represented as a superposition of the EM vectors with non-negative EMCs as follows:

v=∑i=1mλiei.
 MathType@MTEF@5@5@+=feaafiart1ev1aqatCvAUfKttLearuWrP9MDH5MBPbIqV92AaeXatLxBI9gBaebbnrfifHhDYfgasaacH8akY=wiFfYdH8Gipec8Eeeu0xXdbba9frFj0=OqFfea0dXdd9vqai=hGuQ8kuc9pgc9s8qqaq=dirpe0xb9q8qiLsFr0=vr0=vr0dc8meaabaqaciaacaGaaeqabaqabeGadaaakeaacqWH2bGDcqGH9aqpdaaeWbqaaGGaciab=T7aSnaaBaaaleaacqWGPbqAaeqaaOGaeCyzau2aaSbaaSqaaiabdMgaPbqabaaabaGaemyAaKMaeyypa0JaeGymaedabaGaemyBa0ganiabggHiLdGccqGGUaGlaaa@3D2F@

#### EMC spectrum for wild-type

Generally the EMCs are not uniquely determined. For FBA, an objective function can be designed for phenomenological behaviors such as cell growth and product formation to determine the solution spectra of EMCs. Here we do not define any specific objective function involving phenomenological behaviors. Based on the α-spectrum [10], the allowable solution space of the EMCs is calculated by maximizing or minimizing each EMC in a given steady-state flux distribution as follows:

for *j *= *1, 2, ..., m*

*Maximize λ*_*j*_, subject to **v = P·c**, *λ*_*j *_≥ 0 (*j *= *1, 2, ..., m*),

for *j *= *1, 2, ..., m*

*Minimize λ*_*j*_, subject to **v = P·c**, *λ*_*j *_≥ 0 (*j *= *1, 2, ..., m*).

Linear programming is performed using Matlab (Mathworks Inc., Natick, MA) to optimize the EMC vectors for wild type. For each *j *in Eqs. (5, 6), as the EMC vectors (**c**), **r**_2*j*-1 _= (*γ*_1,2*j*-1_, *γ*_2,2*j*-1_, ..., *γ*_*m*,2*j*-1_)^*t*^and **r**_2*j *_= (*γ*_1,2*j*_, *γ*_2,2*j*_, ..., *γ*_*m*,2*j*_)^*t *^are obtained. Consequently, *2m *of the EMC vectors for wild type: **r**_*j *_= (*γ*_1*j*_, *γ*_2*j*_, ..., *γ*_*mj*_)^*t *^(*j *= *1, 2, ..., 2m*) are generated, where *γ*_*ij *_is the *i*-th EMC value of the *j*-th EMC vector.

#### Enzyme-Control Flux (ECF)

In the ECF model, the power law formula integrates the change in an enzyme activity profile between wild-type and a mutant into the EMCs of wild-type to calculate the EMCs of the mutant, thereby simulating the flux distribution of the mutant.

First, the ECF model is constructed based on the correlation equation between the intermediate EMC vectors for a mutant:

inter_rjmutant=(interγ1,jmutant,interγ2,jmutant,...,interγm,jmutant)t(j=1,2,...,2m),
 MathType@MTEF@5@5@+=feaafiart1ev1aaatCvAUfKttLearuWrP9MDH5MBPbIqV92AaeXatLxBI9gBaebbnrfifHhDYfgasaacH8akY=wiFfYdH8Gipec8Eeeu0xXdbba9frFj0=OqFfea0dXdd9vqai=hGuQ8kuc9pgc9s8qqaq=dirpe0xb9q8qiLsFr0=vr0=vr0dc8meaabaqaciaacaGaaeqabaqabeGadaaakeaafaqabeqacaaabaGaeCyAaKMaeCOBa4MaeCiDaqNaeCyzauMaeCOCaiNaeC4xa8LaeCOCai3aa0baaSqaaiabdQgaQbqaaiabd2gaTjabdwha1jabdsha0jabdggaHjabd6gaUjabdsha0baakiabg2da9iabcIcaOiabdMgaPjabd6gaUjabdsha0jabdwgaLjabdkhaYHGaciab=n7aNnaaDaaaleaacqqGXaqmcqqGSaalieGacqGFQbGAaeaacqGFTbqBcqGF1bqDcqGF0baDcqGFHbqycqGFUbGBcqGF0baDaaGccqGGSaalcqWGPbqAcqWGUbGBcqWG0baDcqWGLbqzcqWGYbGCcqWFZoWzdaqhaaWcbaGaeeOmaiJaeeilaWIae4NAaOgabaGae4xBa0Mae4xDauNae4hDaqNae4xyaeMae4NBa4Mae4hDaqhaaOGaeiilaWIaeiOla4IaeiOla4IaeiOla4IaeiilaWIaemyAaKMaemOBa4MaemiDaqNaemyzauMaemOCaiNae83SdC2aa0baaSqaaiab+1gaTjabbYcaSiab+PgaQbqaaiab+1gaTjab+vha1jab+rha0jab+fgaHjab+5gaUjab+rha0baakiabcMcaPmaaCaaaleqabaGaemiDaqhaaaGcbaGaeiikaGIaemOAaOMaeyypa0JaemymaeJaemilaWIaemOmaiJaemilaWIaemOla4IaemOla4IaemOla4IaemilaWIaemOmaiJaemyBa0MaeiykaKcaaiabcYcaSaaa@95BB@

and the EMC vectors of wild-type **r**_*j*_. The superscript of *mutant *indicates a mutant. EMCs indicate the weight of flux through their corresponding EMs, which is affected by the enzyme activities that belong to the EM. Generally, Metabolic Control Analysis (MCA) says that control of metabolic flux is not determined by a rate-limiting step but shared in many enzyme reactions. In analogy to MCA, we assume that the fluxes through EMs are shared in the enzyme reactions that belong to the EMs. On the other hand, it is difficult to identify the degree by which each enzyme affects the EMC involving it. Thus, assuming that changes in the enzyme activities that belong to an EM affect the flux through the EM synergistically, the power-law formalism provides the correlation between inter_rjmutant
 MathType@MTEF@5@5@+=feaafiart1ev1aaatCvAUfKttLearuWrP9MDH5MBPbIqV92AaeXatLxBI9gBaebbnrfifHhDYfgasaacH8akY=wiFfYdH8Gipec8Eeeu0xXdbba9frFj0=OqFfea0dXdd9vqai=hGuQ8kuc9pgc9s8qqaq=dirpe0xb9q8qiLsFr0=vr0=vr0dc8meaabaqaciaacaGaaeqabaqabeGadaaakeaacqWHPbqAcqWHUbGBcqWH0baDcqWHLbqzcqWHYbGCcqWHFbWxcqWHYbGCdaqhaaWcbaGaemOAaOgabaGaemyBa0MaemyDauNaemiDaqNaemyyaeMaemOBa4MaemiDaqhaaaaa@405F@(interγijmutant
 MathType@MTEF@5@5@+=feaafiart1ev1aaatCvAUfKttLearuWrP9MDH5MBPbIqV92AaeXatLxBI9gBaebbnrfifHhDYfgasaacH8akY=wiFfYdH8Gipec8Eeeu0xXdbba9frFj0=OqFfea0dXdd9vqai=hGuQ8kuc9pgc9s8qqaq=dirpe0xb9q8qiLsFr0=vr0=vr0dc8meaabaqaciaacaGaaeqabaqabeGadaaakeaacqWGPbqAcqWGUbGBcqWG0baDcqWGLbqzcqWGYbGCiiGacqWFZoWzdaqhaaWcbaacbiGae4xAaKMae4NAaOgabaGae4xBa0Mae4xDauNae4hDaqNae4xyaeMae4NBa4Mae4hDaqhaaaaa@407B@) and **r**_*j*_(*γ*_*ij*_):

inter_rjmutant=(γ1j∏p=1nαp1βγ2j∏p=1nαp2β...γmj∏p=1nαpmβ) (j=1,2,...,2m),
 MathType@MTEF@5@5@+=feaafiart1ev1aaatCvAUfKttLearuWrP9MDH5MBPbIqV92AaeXatLxBI9gBaebbnrfifHhDYfgasaacH8akY=wiFfYdH8Gipec8Eeeu0xXdbba9frFj0=OqFfea0dXdd9vqai=hGuQ8kuc9pgc9s8qqaq=dirpe0xb9q8qiLsFr0=vr0=vr0dc8meaabaqaciaacaGaaeqabaqabeGadaaakeaafaqabeqacaaabaGaeCyAaKMaeCOBa4MaeCiDaqNaeCyzauMaeCOCaiNaeC4xa8LaeCOCai3aa0baaSqaaiabdQgaQbqaaiabd2gaTjabdwha1jabdsha0jabdggaHjabd6gaUjabdsha0baakiaam2dadaqadaqaauaabeqaeeaaaaqaaiabeo7aNnaaBaaaleaacqqGXaqmieGacqWFQbGAaeqaaOWaaebCaeaaiiGacqGFXoqydaqhaaWcbaGaemiCaaNaemymaedabaGae4NSdigaaaqaaiabdchaWjabg2da9iabigdaXaqaaiabd6gaUbqdcqGHpis1aaGcbaGaeq4SdC2aaSbaaSqaaiabbkdaYiab=PgaQbqabaGcdaqeWbqaaiab+f7aHnaaDaaaleaacqWGWbaCcqaIYaGmaeaacqGFYoGyaaaabaGaemiCaaNaeyypa0JaeGymaedabaGaemOBa4ganiabg+GivdaakeaacqGGUaGlcqGGUaGlcqGGUaGlaeaacqaHZoWzdaWgaaWcbaGae8xBa0Mae8NAaOgabeaakmaarahabaGae4xSde2aa0baaSqaaiabdchaWjabd2gaTbqaaiab+j7aIbaaaeaacqWGWbaCcqGH9aqpcqaIXaqmaeaacqWGUbGBa0Gaey4dIunaaaaakiaawIcacaGLPaaaaeaacqqGGaaicqqGOaakcqWGQbGAcqGH9aqpcqWGXaqmcqWGSaalcqWGYaGmcqWGSaalcqWGUaGlcqWGUaGlcqWGUaGlcqWGSaalcqWGYaGmcqWGTbqBcqGGPaqkaaGaeiilaWcaaa@8822@

or

interγijmutant=γij∏p=1nαpiβ,(i=1,2,..,m;  j=1,2,...,2m)
 MathType@MTEF@5@5@+=feaafiart1ev1aaatCvAUfKttLearuWrP9MDH5MBPbIqV92AaeXatLxBI9gBaebbnrfifHhDYfgasaacH8akY=wiFfYdH8Gipec8Eeeu0xXdbba9frFj0=OqFfea0dXdd9vqai=hGuQ8kuc9pgc9s8qqaq=dirpe0xb9q8qiLsFr0=vr0=vr0dc8meaabaqaciaacaGaaeqabaqabeGadaaakeaafaqabeqacaaabaGaemyAaKMaemOBa4MaemiDaqNaemyzauMaemOCaihcciGae83SdC2aa0baaSqaaGqaciab+LgaPjab+PgaQbqaaiab+1gaTjab+vha1jab+rha0jab+fgaHjab+5gaUjab+rha0baakiabg2da9iab=n7aNnaaBaaaleaacqGFPbqAcqGFQbGAaeqaaOWaaebCaeaacqWFXoqydaqhaaWcbaGaemiCaaNaemyAaKgabaGae8NSdigaaaqaaiabdchaWjabg2da9iabigdaXaqaaiabd6gaUbqdcqGHpis1aOGaeiilaWcabaGaeiikaGIaemyAaKMaeyypa0JaemymaeJaemilaWIaemOmaiJaemilaWIaemOla4IaemOla4IaemilaWIaemyBa0Maei4oaSJaeeiiaaIaeeiiaaIaemOAaOMaeyypa0JaemymaeJaemilaWIaemOmaiJaemilaWIaemOla4IaemOla4IaemOla4IaemilaWIaemOmaiJaemyBa0MaeiykaKcaaaaa@6E3D@

where β is the power factor. The symbol of *α*_*pi *_is defined by:

αpi={ap(epi≠0)1(epi=0).
 MathType@MTEF@5@5@+=feaafiart1ev1aaatCvAUfKttLearuWrP9MDH5MBPbIqV92AaeXatLxBI9gBaebbnrfifHhDYfgasaacH8akY=wiFfYdH8Gipec8Eeeu0xXdbba9frFj0=OqFfea0dXdd9vqai=hGuQ8kuc9pgc9s8qqaq=dirpe0xb9q8qiLsFr0=vr0=vr0dc8meaabaqaciaacaGaaeqabaqabeGadaaakeaaiiGacqWFXoqydaWgaaqcbaAaaiabdchaWjabdMgaPbqabaGccqGH9aqpdaGabaqaauaabeqaciaaaeaacqWGHbqydaWgaaWcbaacbiGae4hCaahabeaaaOqaaiabcIcaOiab+vgaLnaaBaaaleaacqGFWbaCcqGFPbqAaeqaaOGaeyiyIKRaeGimaaJaeiykaKcabaGaeGymaedabaGaeiikaGIae4xzau2aaSbaaSqaaiab+bhaWjab+LgaPbqabaGccqGH9aqpcqaIWaamcqGGPaqkaaaacaGL7baacqGGUaGlaaa@4968@

where *α*_*p *_is the relative enzyme activity of a mutant to wild-type for the *p*-th flux. The power factor of β is the unique parameter that adjusts ECF to a real metabolic system. For simplification, the power factor is set as the same value for each enzyme activity. If a given enzyme activity is zero, the EMC involving it is zero according to the power-law formula.

**Figure 2:**
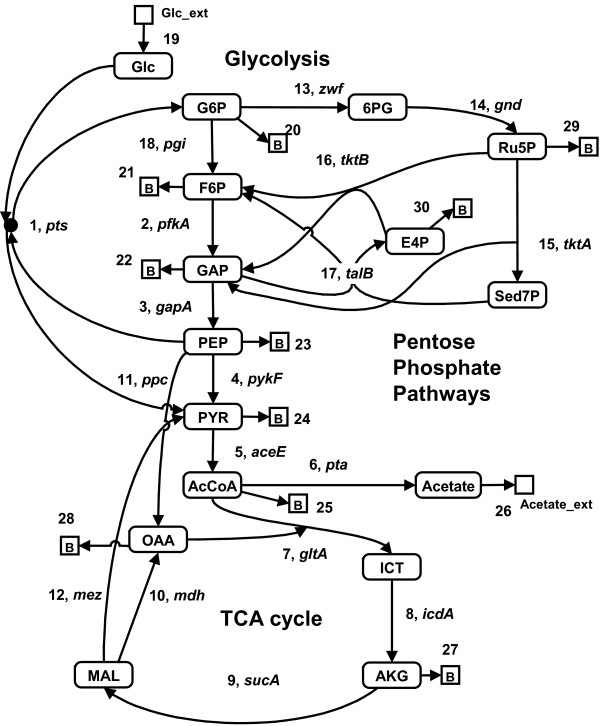
**A schematic diagram of central metabolism for *E. coli***. The network map is made based on our previous experiments. The numbers in the figure are those of reactions shown in Table 1. The gene name is representative of the genes related to the reaction. The metabolites indicated by squares, i.e., B (biomass), Glc_ext (environmental glucose) and Acetate_ext (environmental acetate), are external. All other metabolites, enclosed by rounded rectangles, are internal.

Second, the EMC vector for the mutant, as defined by:

rjmutant=(γ1,jmutant,γ2,jmutant,...,γm,jmutant)t(j=1,2,...,2m),
 MathType@MTEF@5@5@+=feaafiart1ev1aaatCvAUfKttLearuWrP9MDH5MBPbIqV92AaeXatLxBI9gBaebbnrfifHhDYfgasaacH8akY=wiFfYdH8Gipec8Eeeu0xXdbba9frFj0=OqFfea0dXdd9vqai=hGuQ8kuc9pgc9s8qqaq=dirpe0xb9q8qiLsFr0=vr0=vr0dc8meaabaqaciaacaGaaeqabaqabeGadaaakeGabaaSbuaabeqabiaaaeaacqWHYbGCdaqhaaWcbaGaemOAaOgabaGaemyBa0MaemyDauNaemiDaqNaemyyaeMaemOBa4MaemiDaqhaaOGaeyypa0JaeiikaGccciGae83SdC2aa0baaSqaaiabbgdaXiabbYcaSGqaciab+PgaQbqaaiab+1gaTjab+vha1jab+rha0jab+fgaHjab+5gaUjab+rha0baakiabcYcaSiab=n7aNnaaDaaaleaacqqGYaGmcqqGSaalcqGFQbGAaeaacqGFTbqBcqGF1bqDcqGF0baDcqGFHbqycqGFUbGBcqGF0baDaaGccqGGSaalcqGGUaGlcqGGUaGlcqGGUaGlcqGGSaalcqWFZoWzdaqhaaWcbaGae4xBa0MaeeilaWIae4NAaOgabaGae4xBa0Mae4xDauNae4hDaqNae4xyaeMae4NBa4Mae4hDaqhaaOGaeiykaKYaaWbaaSqabeaacqWG0baDaaaakeaacqGGOaakcqWGQbGAcqGH9aqpcqWGXaqmcqWGSaalcqWGYaGmcqWGSaalcqWGUaGlcqWGUaGlcqWGUaGlcqWGSaalcqWGYaGmcqWGTbqBcqGGPaqkaaGaeiilaWcaaa@794C@

is mathematically connected to inter_rjmutant
 MathType@MTEF@5@5@+=feaafiart1ev1aaatCvAUfKttLearuWrP9MDH5MBPbIqV92AaeXatLxBI9gBaebbnrfifHhDYfgasaacH8akY=wiFfYdH8Gipec8Eeeu0xXdbba9frFj0=OqFfea0dXdd9vqai=hGuQ8kuc9pgc9s8qqaq=dirpe0xb9q8qiLsFr0=vr0=vr0dc8meaabaqaciaacaGaaeqabaqabeGadaaakeaacqWHPbqAcqWHUbGBcqWH0baDcqWHLbqzcqWHYbGCcqWHFbWxcqWHYbGCdaqhaaWcbaGaemOAaOgabaGaemyBa0MaemyDauNaemiDaqNaemyyaeMaemOBa4MaemiDaqhaaaaa@405F@ under the constraint that the incoming flux (*v*_*q*_) is setting to 100 (e.g., *v*_19 _= 100 in Figure 2) as follows:

rjmutant=vquq⋅(P⋅inter_rjmutant)inter_rjmutant(j=1,2,...,2m).
 MathType@MTEF@5@5@+=feaafiart1ev1aaatCvAUfKttLearuWrP9MDH5MBPbIqV92AaeXatLxBI9gBaebbnrfifHhDYfgasaacH8akY=wiFfYdH8Gipec8Eeeu0xXdbba9frFj0=OqFfea0dXdd9vqai=hGuQ8kuc9pgc9s8qqaq=dirpe0xb9q8qiLsFr0=vr0=vr0dc8meaabaqaciaacaGaaeqabaqabeGadaaakeaafaqabeqacaaabaGaeCOCai3aa0baaSqaaGqaciab=PgaQbqaaiab=1gaTjab=vha1jab=rha0jab=fgaHjab=5gaUjab=rha0baakiabg2da9maalaaabaGaemODay3aaSbaaSqaaiabdghaXbqabaaakeaacqWH1bqDdaWgaaWcbaGaemyCaehabeaakiabgwSixlabcIcaOiabhcfaqjabgwSixlabhMgaPjabh6gaUjabhsha0jabhwgaLjabhkhaYjabh+faFjabhkhaYnaaDaaaleaacqWFQbGAaeaacqWFTbqBcqWF1bqDcqWF0baDcqWFHbqycqWFUbGBcqWF0baDaaGccqGGPaqkaaGaeCyAaKMaeCOBa4MaeCiDaqNaeCyzauMaeCOCaiNaeC4xa8LaeCOCai3aa0baaSqaaiab=PgaQbqaaiab=1gaTjab=vha1jab=rha0jab=fgaHjab=5gaUjab=rha0baaaOqaaiabcIcaOiabdQgaQjabg2da9iabdgdaXiabdYcaSiabdkdaYiabdYcaSiabd6caUiabd6caUiabd6caUiabdYcaSiabdkdaYiabd2gaTjabcMcaPaaacqGGUaGlaaa@7C81@

or

γijmutant=vquq⋅(P⋅inter_rjmutant)interγijmutant(i=1,2,..,m;  j=1,2,...,2m),
 MathType@MTEF@5@5@+=feaafiart1ev1aaatCvAUfKttLearuWrP9MDH5MBPbIqV92AaeXatLxBI9gBaebbnrfifHhDYfgasaacH8akY=wiFfYdH8Gipec8Eeeu0xXdbba9frFj0=OqFfea0dXdd9vqai=hGuQ8kuc9pgc9s8qqaq=dirpe0xb9q8qiLsFr0=vr0=vr0dc8meaabaqaciaacaGaaeqabaqabeGadaaakeaafaqabeqacaaabaacciGae83SdC2aa0baaSqaaGqaciab+LgaPjab+PgaQbqaaiab+1gaTjab+vha1jab+rha0jab+fgaHjab+5gaUjab+rha0baakiabg2da9maalaaabaGaemODay3aaSbaaSqaaiabdghaXbqabaaakeaacqWH1bqDdaWgaaWcbaGaemyCaehabeaakiabgwSixlabcIcaOiabhcfaqjabgwSixlabhMgaPjabh6gaUjabhsha0jabhwgaLjabhkhaYjabh+faFjabhkhaYnaaDaaaleaacqGFQbGAaeaacqGFTbqBcqGF1bqDcqGF0baDcqGFHbqycqGFUbGBcqGF0baDaaGccqGGPaqkaaGaemyAaKMaemOBa4MaemiDaqNaemyzauMaemOCaiNae83SdC2aa0baaSqaaiab+LgaPjab+PgaQbqaaiab+1gaTjab+vha1jab+rha0jab+fgaHjab+5gaUjab+rha0baaaOqaaiabcIcaOiabdMgaPjabg2da9iabdgdaXiabdYcaSiabdkdaYiabdYcaSiabd6caUiabd6caUiabdYcaSiabd2gaTjabcUda7iabbccaGiabbccaGiabdQgaQjabg2da9iabdgdaXiabdYcaSiabdkdaYiabdYcaSiabd6caUiabd6caUiabd6caUiabdYcaSiabdkdaYiabd2gaTjabcMcaPaaacqGGSaalaaa@8AB8@

Here, **u**_*q *_is the *m*-dimensional raw unit vector with the *q*-th component of 1.

Finally, the flux distribution of the mutant vjmutant=(v1jmutant,v2jmutant,...,vnjmutant)t
 MathType@MTEF@5@5@+=feaafiart1ev1aaatCvAUfKttLearuWrP9MDH5MBPbIqV92AaeXatLxBI9gBaebbnrfifHhDYfgasaacH8akY=wiFfYdH8Gipec8Eeeu0xXdbba9frFj0=OqFfea0dXdd9vqai=hGuQ8kuc9pgc9s8qqaq=dirpe0xb9q8qiLsFr0=vr0=vr0dc8meaabaqaciaacaGaaeqabaqabeGadaaakeaacqWH2bGDdaqhaaWcbaGaemOAaOgabaGaemyBa0MaemyDauNaemiDaqNaemyyaeMaemOBa4MaemiDaqhaaOGaeyypa0JaeiikaGIaemODay3aa0baaSqaaiabigdaXiabdQgaQbqaaiabd2gaTjabdwha1jabdsha0jabdggaHjabd6gaUjabdsha0baakiabcYcaSiabdAha2naaDaaaleaacqaIYaGmcqWGQbGAaeaacqWGTbqBcqWG1bqDcqWG0baDcqWGHbqycqWGUbGBcqWG0baDaaGccqGGSaalcqGGUaGlcqGGUaGlcqGGUaGlcqGGSaalcqWG2bGDdaqhaaWcbaGaemOBa4MaemOAaOgabaGaemyBa0MaemyDauNaemiDaqNaemyyaeMaemOBa4MaemiDaqhaaOGaeiykaKYaaWbaaSqabeaacqWG0baDaaaaaa@675D@ is provided by:

vjmutant=P⋅rjmutant(j=1,2,...,2m).
 MathType@MTEF@5@5@+=feaafiart1ev1aaatCvAUfKttLearuWrP9MDH5MBPbIqV92AaeXatLxBI9gBaebbnrfifHhDYfgasaacH8akY=wiFfYdH8Gipec8Eeeu0xXdbba9frFj0=OqFfea0dXdd9vqai=hGuQ8kuc9pgc9s8qqaq=dirpe0xb9q8qiLsFr0=vr0=vr0dc8meaabaqaciaacaGaaeqabaqabeGadaaakeaafaqabeqacaaabaGaeCODay3aa0baaSqaaiabdQgaQbqaaiabd2gaTjabdwha1jabdsha0jabdggaHjabd6gaUjabdsha0baaiiqakiab=1da9iabhcfaqjabgwSixlabhkhaYnaaDaaaleaacqWGQbGAaeaacqWGTbqBcqWG1bqDcqWG0baDcqWGHbqycqWGUbGBcqWG0baDaaaakeaacqGGOaakcqWGQbGAcqGH9aqpcqWGXaqmcqWGSaalcqWGYaGmcqWGSaalcqWGUaGlcqWGUaGlcqWGUaGlcqWGSaalcqWGYaGmcqWGTbqBcqGGPaqkaaGaeiOla4caaa@568F@

The ECF model does not use any kinetics to calculate the steady-state flux distributions for mutants. In this context ECF is a static or non-mechanistic model that links an enzyme activity profile to a metabolic flux distribution.

#### Characterization of ECF for an enzyme activity profile

To demonstrate the validity of ECF, ECF is applied to simulate the flux distribution of a mutant by integrating enzyme activity data into the EMC vector of wild type. Since a distribution occurs in the simulated flux vectors of {vjmutant|j=1,2,...,2m}
 MathType@MTEF@5@5@+=feaafiart1ev1aaatCvAUfKttLearuWrP9MDH5MBPbIqV92AaeXatLxBI9gBaebbnrfifHhDYfgasaacH8akY=wiFfYdH8Gipec8Eeeu0xXdbba9frFj0=OqFfea0dXdd9vqai=hGuQ8kuc9pgc9s8qqaq=dirpe0xb9q8qiLsFr0=vr0=vr0dc8meaabaqaciaacaGaaeqabaqabeGadaaakeaacqGG7bWEcqWH2bGDdaqhaaWcbaGaemOAaOgabaGaemyBa0MaemyDauNaemiDaqNaemyyaeMaemOBa4MaemiDaqhaaOGaeiiFaWNaemOAaOMaeyypa0JaeGymaeJaeiilaWIaeGOmaiJaeiilaWIaeiOla4IaeiOla4IaeiOla4IaeiilaWIaeGOmaiJaemyBa0MaeiyFa0haaa@4887@ in Eq. (12), the mean and the relative standard deviation are calculated for each flux. The mean simulated flux distribution of vmeanmutant
 MathType@MTEF@5@5@+=feaafiart1ev1aaatCvAUfKttLearuWrP9MDH5MBPbIqV92AaeXatLxBI9gBaebbnrfifHhDYfgasaacH8akY=wiFfYdH8Gipec8Eeeu0xXdbba9frFj0=OqFfea0dXdd9vqai=hGuQ8kuc9pgc9s8qqaq=dirpe0xb9q8qiLsFr0=vr0=vr0dc8meaabaqaciaacaGaaeqabaqabeGadaaakeaacqWH2bGDdaqhaaWcbaGaemyBa0MaemyzauMaemyyaeMaemOBa4gabaGaemyBa0MaemyDauNaemiDaqNaemyyaeMaemOBa4MaemiDaqhaaaaa@3C20@ is defined by:

vmeanmutant=12m∑j=12mvjmutant
 MathType@MTEF@5@5@+=feaafiart1ev1aaatCvAUfKttLearuWrP9MDH5MBPbIqV92AaeXatLxBI9gBaebbnrfifHhDYfgasaacH8akY=wiFfYdH8Gipec8Eeeu0xXdbba9frFj0=OqFfea0dXdd9vqai=hGuQ8kuc9pgc9s8qqaq=dirpe0xb9q8qiLsFr0=vr0=vr0dc8meaabaqaciaacaGaaeqabaqabeGadaaakeaacqWH2bGDdaqhaaWcbaGaemyBa0MaemyzauMaemyyaeMaemOBa4gabaGaemyBa0MaemyDauNaemiDaqNaemyyaeMaemOBa4MaemiDaqhaaOGaeyypa0ZaaSaaaeaacqaIXaqmaeaacqaIYaGmcqWGTbqBaaWaaabCaeaacqWH2bGDdaqhaaWcbaGaemOAaOgabaGaemyBa0MaemyDauNaemiDaqNaemyyaeMaemOBa4MaemiDaqhaaaqaaiabdQgaQjabg2da9iabigdaXaqaaiabikdaYiabd2gaTbqdcqGHris5aaaa@53CF@

or

vi meanmutant=12m∑j=12mvi jmutant(i=1,2,..n)
 MathType@MTEF@5@5@+=feaafiart1ev1aaatCvAUfKttLearuWrP9MDH5MBPbIqV92AaeXatLxBI9gBaebbnrfifHhDYfgasaacH8akY=wiFfYdH8Gipec8Eeeu0xXdbba9frFj0=OqFfea0dXdd9vqai=hGuQ8kuc9pgc9s8qqaq=dirpe0xb9q8qiLsFr0=vr0=vr0dc8meaabaqaciaacaGaaeqabaqabeGadaaakeaafaqabeqacaaabaGaemODay3aa0baaSqaaiabdMgaPjabbccaGiabd2gaTjabdwgaLjabdggaHjabd6gaUbqaaiabd2gaTjabdwha1jabdsha0jabdggaHjabd6gaUjabdsha0baakiabg2da9maalaaabaGaeGymaedabaGaeGOmaiJaemyBa0gaamaaqahabaGaemODay3aa0baaSqaaiabdMgaPjabbccaGiabdQgaQbqaaiabd2gaTjabdwha1jabdsha0jabdggaHjabd6gaUjabdsha0baaaeaacqWGQbGAcqGH9aqpcqaIXaqmaeaacqaIYaGmcqWGTbqBa0GaeyyeIuoaaOqaaiabcIcaOiabdMgaPjabg2da9iabdgdaXiabdYcaSiabdkdaYiabdYcaSiabd6caUiabd6caUiabd6gaUjabcMcaPaaaaaa@62FE@

The relative model error for the *i*-th flux is defined by:

Relative Model Error for Flux (i) =|vi meanmutant - vi expmutant|vi expmutant(i=1,2,..n)
 MathType@MTEF@5@5@+=feaafiart1ev1aqatCvAUfKttLearuWrP9MDH5MBPbIqV92AaeXatLxBI9gBaebbnrfifHhDYfgasaacH8akY=wiFfYdH8Gipec8Eeeu0xXdbba9frFj0=OqFfea0dXdd9vqai=hGuQ8kuc9pgc9s8qqaq=dirpe0xb9q8qiLsFr0=vr0=vr0dc8meaabaqaciaacaGaaeqabaqabeGadaaakeaafaqabeqacaaabaGaeeOuaiLaeeyzauMaeeiBaWMaeeyyaeMaeeiDaqNaeeyAaKMaeeODayNaeeyzauMaeeiiaaIaeeyta0Kaee4Ba8MaeeizaqMaeeyzauMaeeiBaWMaeeiiaaIaeeyrauKaeeOCaiNaeeOCaiNaee4Ba8MaeeOCaiNaeeiiaaIaeeOzayMaee4Ba8MaeeOCaiNaeeiiaaIaeeOrayKaeeiBaWMaeeyDauNaeeiEaGNaeeiiaaIaeeikaGIaemyAaKMaeeykaKIaeeiiaacccaGae8xpa0ZaaSaaaeaadaabdaqaaiabdAha2naaDaaaleaacqWGPbqAcqqGGaaicqWGTbqBcqWGLbqzcqWGHbqycqWGUbGBaeaacqWGTbqBcqWG1bqDcqWG0baDcqWGHbqycqWGUbGBcqWG0baDaaGccqqGGaaicqqGTaqlcqqGGaaicqWG2bGDdaqhaaWcbaGaemyAaKMaeeiiaaIaemyzauMaemiEaGNaemiCaahabaGaemyBa0MaemyDauNaemiDaqNaemyyaeMaemOBa4MaemiDaqhaaaGccaGLhWUaayjcSdaabaGaemODay3aa0baaSqaaiabdMgaPjabbccaGiabdwgaLjabdIha4jabdchaWbqaaiabd2gaTjabdwha1jabdsha0jabdggaHjabd6gaUjabdsha0baaaaaakeaacqGGOaakcqWGPbqAcqGH9aqpcqWGXaqmcqWGSaalcqWGYaGmcqWGSaalcqWGUaGlcqWGUaGlcqWGUbGBcqGGPaqkaaaaaa@99D0@

where vi expmutant
 MathType@MTEF@5@5@+=feaafiart1ev1aqatCvAUfKttLearuWrP9MDH5MBPbIqV92AaeXatLxBI9gBaebbnrfifHhDYfgasaacH8akY=wiFfYdH8Gipec8Eeeu0xXdbba9frFj0=OqFfea0dXdd9vqai=hGuQ8kuc9pgc9s8qqaq=dirpe0xb9q8qiLsFr0=vr0=vr0dc8meaabaqaciaacaGaaeqabaqabeGadaaakeaacqWG2bGDdaqhaaWcbaGaemyAaKMaeeiiaaIaemyzauMaemiEaGNaemiCaahabaGaemyBa0MaemyDauNaemiDaqNaemyyaeMaemOBa4MaemiDaqhaaaaa@3D0E@ is the *i*-th flux for an experimental mutant. The subscript of "exp" means experimental data.

To characterize the overall accuracy of ECF, the model error, which is the difference between the estimated flux distribution and the experimental one for each optimized EM vector, is defined by:

Model Error(j)=1n∑i=1n(vijmutant−vi expmutant)2(j=1,2,...,2m).
 MathType@MTEF@5@5@+=feaafiart1ev1aaatCvAUfKttLearuWrP9MDH5MBPbIqV92AaeXatLxBI9gBaebbnrfifHhDYfgasaacH8akY=wiFfYdH8Gipec8Eeeu0xXdbba9frFj0=OqFfea0dXdd9vqai=hGuQ8kuc9pgc9s8qqaq=dirpe0xb9q8qiLsFr0=vr0=vr0dc8meaabaqaciaacaGaaeqabaqabeGadaaakeaafaqabeqacaaabaGaeeyta0Kaee4Ba8MaeeizaqMaeeyzauMaeeiBaWMaeeiiaaIaeeyrauKaeeOCaiNaeeOCaiNaee4Ba8MaeeOCaiNaeeikaGIaemOAaOMaeeykaKIaeyypa0ZaaOaaaeaadaWcaaqaaiabigdaXaqaaiabd6gaUbaadaaeWbqaaiabcIcaOiabdAha2naaDaaaleaacqWGPbqAcqWGQbGAaeaacqWGTbqBcqWG1bqDcqWG0baDcqWGHbqycqWGUbGBcqWG0baDaaGccqGHsislcqWG2bGDdaqhaaWcbaGaemyAaKMaeeiiaaIaemyzauMaemiEaGNaemiCaahabaGaemyBa0MaemyDauNaemiDaqNaemyyaeMaemOBa4MaemiDaqhaaaqaaiabdMgaPjabg2da9iabigdaXaqaaiabd6gaUbqdcqGHris5aOGaeiykaKYaaWbaaSqabeaacqaIYaGmaaaabeaaaOqaaiabcIcaOiabdQgaQjabg2da9iabdgdaXiabdYcaSiabdkdaYiabdYcaSiabd6caUiabd6caUiabd6caUiabdYcaSiabdkdaYiabd2gaTjabcMcaPaaacqGGUaGlaaa@77A3@

Since the model error has a distribution, the mean and the relative standard deviation are calculated. Model accuracy becomes high as the mean model error decreases.

#### Characterization of ECF for combinatorial enzyme activity profiles

For the metabolic systems employed in this study, enzyme activities are not given for all metabolic reactions. Thus, it raises a question: if the ECF-estimated flux distributions depend on the members of the enzymes incorporated into the model. Therefore, the flux distributions for a mutant need to be simulated for all combinations of the enzymes with measured activities.

The combination number of incorporated enzyme activities (*N*_*c*_) is provided by:

*N*_*c *_= *yCx*,

where *y *is the total number of measured enzymes and *x *is the number of incorporated enzymes in the ECF model. For each combination the flux distributions of {vjmutant|j=1,2,...,2m}
 MathType@MTEF@5@5@+=feaafiart1ev1aaatCvAUfKttLearuWrP9MDH5MBPbIqV92AaeXatLxBI9gBaebbnrfifHhDYfgasaacH8akY=wiFfYdH8Gipec8Eeeu0xXdbba9frFj0=OqFfea0dXdd9vqai=hGuQ8kuc9pgc9s8qqaq=dirpe0xb9q8qiLsFr0=vr0=vr0dc8meaabaqaciaacaGaaeqabaqabeGadaaakeaacqGG7bWEcqWH2bGDdaqhaaWcbaGaemOAaOgabaGaemyBa0MaemyDauNaemiDaqNaemyyaeMaemOBa4MaemiDaqhaaOGaeiiFaWNaemOAaOMaeyypa0JaeGymaeJaeiilaWIaeGOmaiJaeiilaWIaeiOla4IaeiOla4IaeiOla4IaeiilaWIaeGOmaiJaemyBa0MaeiyFa0haaa@4887@ (*j *= *1, 2, ..., 2m*) are simulated. When *x *enzyme activities are incorporated, the number of {vjmutant|j=1,2,...,2m}
 MathType@MTEF@5@5@+=feaafiart1ev1aaatCvAUfKttLearuWrP9MDH5MBPbIqV92AaeXatLxBI9gBaebbnrfifHhDYfgasaacH8akY=wiFfYdH8Gipec8Eeeu0xXdbba9frFj0=OqFfea0dXdd9vqai=hGuQ8kuc9pgc9s8qqaq=dirpe0xb9q8qiLsFr0=vr0=vr0dc8meaabaqaciaacaGaaeqabaqabeGadaaakeaacqGG7bWEcqWH2bGDdaqhaaWcbaGaemOAaOgabaGaemyBa0MaemyDauNaemiDaqNaemyyaeMaemOBa4MaemiDaqhaaOGaeiiFaWNaemOAaOMaeyypa0JaeGymaeJaeiilaWIaeGOmaiJaeiilaWIaeiOla4IaeiOla4IaeiOla4IaeiilaWIaeGOmaiJaemyBa0MaeiyFa0haaa@4887@ is given to:

*L *= *2m*·*yCx*.

Consequently, the flux distribution of a mutant is provided by:

vjmutant=P⋅rjmutant(j=1,2,...,L),
 MathType@MTEF@5@5@+=feaafiart1ev1aaatCvAUfKttLearuWrP9MDH5MBPbIqV92AaeXatLxBI9gBaebbnrfifHhDYfgasaacH8akY=wiFfYdH8Gipec8Eeeu0xXdbba9frFj0=OqFfea0dXdd9vqai=hGuQ8kuc9pgc9s8qqaq=dirpe0xb9q8qiLsFr0=vr0=vr0dc8meaabaqaciaacaGaaeqabaqabeGadaaakeaafaqabeqacaaabaGaeCODay3aa0baaSqaaiabdQgaQbqaaiabd2gaTjabdwha1jabdsha0jabdggaHjabd6gaUjabdsha0baaiiqakiab=1da9iabhcfaqjabgwSixlabhkhaYnaaDaaaleaacqWGQbGAaeaacqWGTbqBcqWG1bqDcqWG0baDcqWGHbqycqWGUbGBcqWG0baDaaaakeaacqGGOaakcqWGQbGAcqGH9aqpcqWGXaqmcqWGSaalcqWGYaGmcqWGSaalcqWGUaGlcqWGUaGlcqWGUaGlcqWGSaalcqWGmbatcqGGPaqkaaGaeiilaWcaaa@555C@

The mean estimated flux of vi meanmutant
 MathType@MTEF@5@5@+=feaafiart1ev1aqatCvAUfKttLearuWrP9MDH5MBPbIqV92AaeXatLxBI9gBaebbnrfifHhDYfgasaacH8akY=wiFfYdH8Gipec8Eeeu0xXdbba9frFj0=OqFfea0dXdd9vqai=hGuQ8kuc9pgc9s8qqaq=dirpe0xb9q8qiLsFr0=vr0=vr0dc8meaabaqaciaacaGaaeqabaqabeGadaaakeaacqWG2bGDdaqhaaWcbaGaemyAaKMaeeiiaaIaemyBa0MaemyzauMaemyyaeMaemOBa4gabaGaemyBa0MaemyDauNaemiDaqNaemyyaeMaemOBa4MaemiDaqhaaaaa@3E3F@ is defined by:

vi meanmutant=1L∑j=1Lvijmutant(i=1,2,..n).
 MathType@MTEF@5@5@+=feaafiart1ev1aaatCvAUfKttLearuWrP9MDH5MBPbIqV92AaeXatLxBI9gBaebbnrfifHhDYfgasaacH8akY=wiFfYdH8Gipec8Eeeu0xXdbba9frFj0=OqFfea0dXdd9vqai=hGuQ8kuc9pgc9s8qqaq=dirpe0xb9q8qiLsFr0=vr0=vr0dc8meaabaqaciaacaGaaeqabaqabeGadaaakeaafaqabeqacaaabaGaemODay3aa0baaSqaaiabdMgaPjabbccaGiabd2gaTjabdwgaLjabdggaHjabd6gaUbqaaiabd2gaTjabdwha1jabdsha0jabdggaHjabd6gaUjabdsha0baakiabg2da9maalaaabaGaeGymaedabaGaemitaWeaamaaqahabaGaemODay3aa0baaSqaaiabdMgaPjabdQgaQbqaaiabd2gaTjabdwha1jabdsha0jabdggaHjabd6gaUjabdsha0baaaeaacqWGQbGAcqGH9aqpcqaIXaqmaeaacqWGmbata0GaeyyeIuoaaOqaaiabcIcaOiabdMgaPjabg2da9iabdgdaXiabdYcaSiabdkdaYiabdYcaSiabd6caUiabd6caUiabd6gaUjabcMcaPaaacqGGUaGlaaa@60B3@

The model error profile with respect to each optimized EM vector is given by:

Model Error(j)=1n∑i=1n(vijmutant−vi expmutant)2(j=1,2,..,L).
 MathType@MTEF@5@5@+=feaafiart1ev1aaatCvAUfKttLearuWrP9MDH5MBPbIqV92AaeXatLxBI9gBaebbnrfifHhDYfgasaacH8akY=wiFfYdH8Gipec8Eeeu0xXdbba9frFj0=OqFfea0dXdd9vqai=hGuQ8kuc9pgc9s8qqaq=dirpe0xb9q8qiLsFr0=vr0=vr0dc8meaabaqaciaacaGaaeqabaqabeGadaaakeaafaqabeqacaaabaGaeeyta0Kaee4Ba8MaeeizaqMaeeyzauMaeeiBaWMaeeiiaaIaeeyrauKaeeOCaiNaeeOCaiNaee4Ba8MaeeOCaiNaeeikaGIaemOAaOMaeeykaKIaeyypa0ZaaOaaaeaadaWcaaqaaiabigdaXaqaaiabd6gaUbaadaaeWbqaaiabcIcaOiabdAha2naaDaaaleaacqWGPbqAcqWGQbGAaeaacqWGTbqBcqWG1bqDcqWG0baDcqWGHbqycqWGUbGBcqWG0baDaaGccqGHsislcqWG2bGDdaqhaaWcbaGaemyAaKMaeeiiaaIaemyzauMaemiEaGNaemiCaahabaGaemyBa0MaemyDauNaemiDaqNaemyyaeMaemOBa4MaemiDaqhaaaqaaiabdMgaPjabg2da9iabigdaXaqaaiabd6gaUbqdcqGHris5aOGaeiykaKYaaWbaaSqabeaacqaIYaGmaaaabeaaaOqaaiabcIcaOiabdQgaQjabg2da9iabdgdaXiabdYcaSiabdkdaYiabdYcaSiabd6caUiabd6caUiabdYcaSiabdYeamjabcMcaPaaacqGGUaGlaaa@758F@

where vi expmutant
 MathType@MTEF@5@5@+=feaafiart1ev1aqatCvAUfKttLearuWrP9MDH5MBPbIqV92AaeXatLxBI9gBaebbnrfifHhDYfgasaacH8akY=wiFfYdH8Gipec8Eeeu0xXdbba9frFj0=OqFfea0dXdd9vqai=hGuQ8kuc9pgc9s8qqaq=dirpe0xb9q8qiLsFr0=vr0=vr0dc8meaabaqaciaacaGaaeqabaqabeGadaaakeaacqWG2bGDdaqhaaWcbaGaemyAaKMaeeiiaaIaemyzauMaemiEaGNaemiCaahabaGaemyBa0MaemyDauNaemiDaqNaemyyaeMaemOBa4MaemiDaqhaaaaa@3D0E@ is the *i*-th experimental flux distribution. Since the model error has a distribution, the mean and the relative standard deviation are calculated. Eq. (21) corresponds to Eq. (16) at *y *= *x*.

### Application example of ECF to a *pykF *knockout mutant model

#### Estimation of EMCs in wild-type

**Figure 3:**
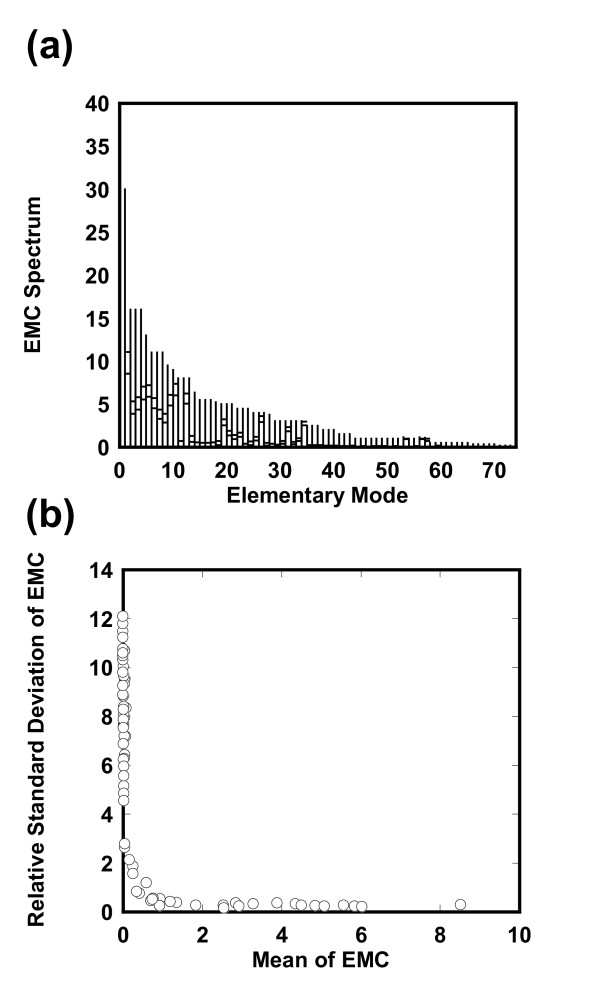
**Estimated EMCs in the *pykF*(-) knockout mutant**. (a) Spectrum for the EMCs with respect to elementary modes. The EMCs with respect to the (*2j-1 *and *2j*)-th optimization trial are calculated by maximizing or minimizing the *j*-th EMC (*j *= *1, 2, ..., 73*). EMs are sorted according to their maximum values. The vertical lines indicate the allowable ranges for the EMCs. Each line has two transverse dashes at the right: The lower one indicates the mean (*m*) and the upper one is the sum of the mean and the standard deviation (σ). (b) Relative standard deviation of the estimated EMC spectrum. The relative standard deviations (σ/*m*) are plotted with respect to the means (*m*). Note that the mean EMCs are not zero.

The *pykF*(-) knockout mutant in *E. coli *is employed to explain the algorithm of ECF (Figure 2 and Table 1)[11] and to demonstrate the feasibility of ECF by applying it to estimation of the flux distribution of the mutant. Elementary mode analysis generated 73 EMs from 30 reactions of the central metabolic network by using FluxAnalyzer [12]. The flux distributions in wild type and the mutant were measured (Table 2). One hundred and forty-six (= 73 × 2) sets of the EMC vectors for wild type **r**_*j *_= (*γ*_1,*j*_, *γ*_2,*j*_, ..., *γ*_*m*,*j*_)^*t *^(*j *= *1, 2, ..., 146*) were optimized by Eqs. (5,6). The resulting EMC vectors showed similar spectrums, i.e., their resultant spectrums do not greatly vary with changes in optimization trials (see Additional file 2). Here we characterized the profiles for the EMC vectors. Figure 3a shows the allowable solution spectrum for each EM, where the spectrum for each EMC contains 146 data points. To explore representative values of each EMC, we calculated the mean (*m*) and standard deviation (*σ*) for the EMC spectrum and plotted the relative standard deviation (*σ/m*) with respect to *m*, as shown in Figure 3b. For small mean values of the EMCs, the relative standard deviations are large, revealing that many data points are scattered apart. In contrast, for large mean values the relative standard deviations are much smaller, showing that most data points are located around the mean.

**Table 1:** Reactions from the *E. coli *central metabolic network

Reaction	Gene name	Enzyme	Chemical reaction
1	*ptsH, ptsI* *crr, glk*	Glucose phosphotransferase system	Glc + PEP => G6P + PYR
2	*pfkA, pfkB* *fba* *tpi*	Phosphofructokinase Fructose-16-bisphosphatate aldolase Triphosphate isomerase	F6P => 2 GAP
3	*gapA* *pgk* *gpmA*	Glycelaldehyde-3-phosphate dehydrogenase Phosphoglycerate kinase Phosphoglycerate mutase I	GAP => PEP
4	*pykF* *pykA*	Pyruvate kinase I	PEP => PYR
5	*aceE* *aceF* *lpdA*	Pyruvate dehydrogenase	PYR => AcCoA
6	*pta, ackA*	Acetyl-CoA synthetase	AcCoA => Acetate
7	*gltA* *acnA, acnB*	Citrate synthase Aconitase A	AcCoA + OAA => ICT
8	*icdA*	Isocitrate dehydrogenase	ICT => AKG
9	*sucA, sucB, lpdA* *sucC, sucD* *sdhC* *fumA, fumB, fumC*	2-ketoglutarate dehydrogenase Succinyl-CoA synthetase Succinate dehydrogenase Fumarase	AKG => MAL
10	*mdh*	Malate dehydrogenase	MAL => OAA
11	*ppc*	Phosphenolpyruvate carboxylase	PEP => OAA
12	*mez*	Malic enzyme	MAL => PYR
13	*zwf*	Glucose-6-phosphate-1-dehydrogenase	G6P => 6PG
14	*gnd*	6-phosphoglycononate dehydrogenase	6PG => Ru5P
	*rpiA*	Ribose-5-phosphate isomerase A	Ru5P => R5P
	*rpe*	Ribose phosphate 3-epimerase	Ru5P => X5P
15	*tktA*	Transketolase I	X5P + R5P => GAP + Sed7P
16	*tktB*	Transketolase II	X5P + E4P => F6P +GAP
17	*talB*	Transaldolase B	GAP + Sed7P => F6P + E4P
18	*pgi*	Phosphoglucoisomerase	G6P => F6P
19		Glucose uptake	Glc_ext => Glc
20		Materials are used for biomass synthesis	G6P => Biomass
21		Materials are used for biomass synthesis	F6P => Biomass
22		Materials are used for biomass synthesis	GAP => Biomass
23		Materials are used for biomass synthesis	PEP =-> Biomass
24		Materials are used for biomass synthesis	PYR => Biomass
25		Materials are used for biomass synthesis	AcCoA => Biomass
26		Membrane transport reaction	Acetate => Acetate_ext
27		Materials are used for biomass synthesis	AKG => Biomass
28		Materials are used for biomass synthesis	OAA => Biomass
29		Materials are used for biomass synthesis	R5P => Biomass
30		Materials are used for biomass synthesis	E4P => Biomass

Abbreviation: AcCoA Acetyl-CoA, AKG α-ketoglutarate, E4P erythrose-4-phosphate, F6P fructose-1,6-bisphosphate, Glc glucose, GAP glyceraldehydes-3-phosphate, G6P glucose-6-phosphate, ICT isocitrate, Mal malate, OAA oxaloacetate, PEP phosphoenolpyruvate, 6PG 6-phosphogluconate, Ru5P ribulose-5-phosphate, X5P, xylulose 5-phosphate, R5P ribose-5-phosphate, Sed7P sedoheptulose-7-phosphate, Glc_ext Glucose external, Acetate_ext Acetate external.

**Table 2:** Experimental data of flux and enzyme activities in wild type and the *pykF*(-) knockout mutant

	Flux in the wild type	Flux in the mutant	Relative enzyme activities in the *pykF*(-) mutant
1	100	100	0.57
2	83	65	0.57
3	163	151	1
4	30	1	0.14
5	107	103	1
6	20	1	0.37
7 (*gltA*)	87	81	0.45
8	87	81	1
9	78	73	1
10	75	52	1.47
11	17	44	2.3
12	3	21	3.5
13	34	79	1.7
14	34	79	1.44
15	11	25	1
16	8	22	1
17	11	25	1
18	65	20	0.59
19	100	100	1
20	1	1	1
21	1	2	1
22	11	1	1
23	16	6	1
24	26	19	1
25	1	21	1
26	19	1	1
27	9	8	1
28	5	15	1
29	4	7	1
30	3	3	1

The cells were cultivated at a dilution rate of 0.1 h^-1^. The glucose uptake flux was normalized to 100. The relative enzyme activities in the mutant were normalized by those in the wild type. Enzymes were extracted from the cultured cells and their activities were measured *in vitro*.

#### Flux estimation in a pykF knockout mutant

**Figure 4:**
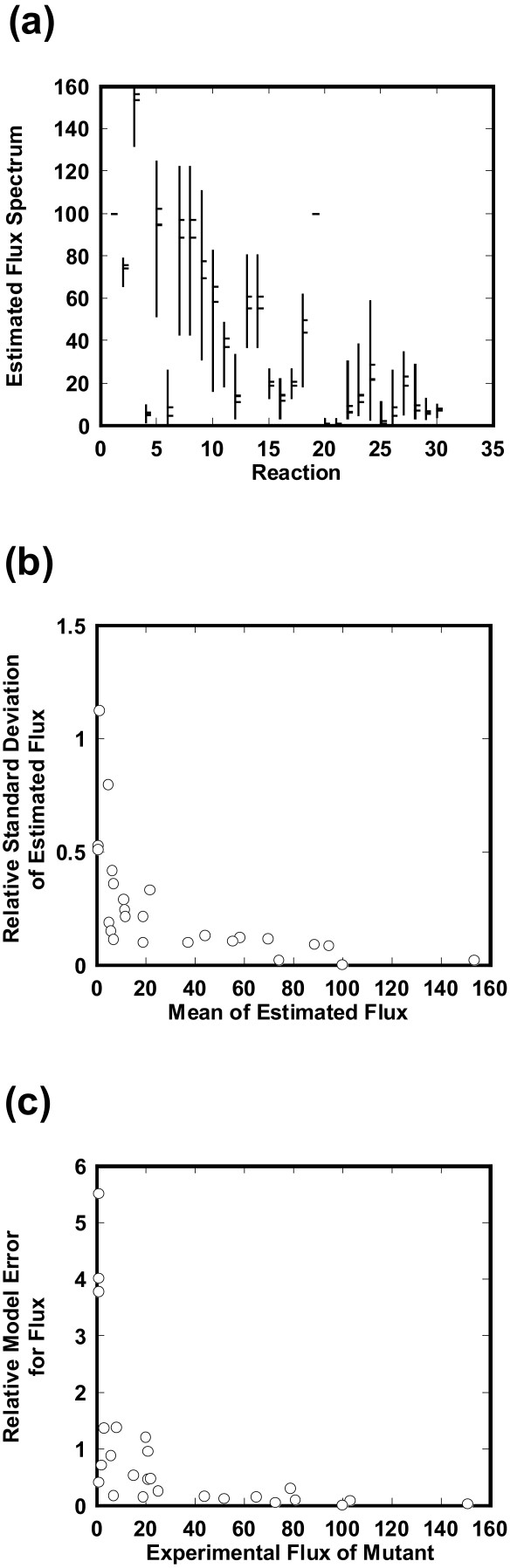
**Characterization of each estimated flux in the *pykF*(-) knockout mutant**. (a) Spectrum for the ECF-simulated metabolic flux in central metabolic pathways of the *pykF*(-) mutant. One hundred forty-six sets of EMCs optimized for wild-type were employed to estimate the flux distribution profiles. The vertical lines indicate the allowable ranges for the estimated flux. Each line has two transverse dashes at the right: The lower one indicates the mean (*m*) and the upper one is the sum of the mean and the standard deviation (σ). (b) Relative standard deviations for the estimated flux spectrum. The relative standard deviations (σ/*m*) are plotted with respect to the means (*m*). **(c) **Relative model error for flux. The relative model error for each flux is plotted with respect to the experimental flux for the mutant.

In this estimation, the factor of β is set to 1, which will be validated in the later section (Validation of the unique parameter of β). Notice that a β factor of 1 does not reflect any linear relationship between flux and each enzyme activity, as shown in Eqs. (10, 11). In order for ECF to simulate the flux distribution in the central metabolic pathways of the *pykF*(-) knockout mutant, we integrated the enzyme activity data (Table 2) into the EMC vectors for wild type according to the power law formalism of Eqs. (7, 8, 9), simulating the EMC vectors for the mutant rjmutant=(γ1,jmutant,γ2,jmutant,...,γm,jmutant)t
 MathType@MTEF@5@5@+=feaafiart1ev1aaatCvAUfKttLearuWrP9MDH5MBPbIqV92AaeXatLxBI9gBaebbnrfifHhDYfgasaacH8akY=wiFfYdH8Gipec8Eeeu0xXdbba9frFj0=OqFfea0dXdd9vqai=hGuQ8kuc9pgc9s8qqaq=dirpe0xb9q8qiLsFr0=vr0=vr0dc8meaabaqaciaacaGaaeqabaqabeGadaaakeGabaaSbiabhkhaYnaaDaaaleaacqWGQbGAaeaacqWGTbqBcqWG1bqDcqWG0baDcqWGHbqycqWGUbGBcqWG0baDaaGccqGH9aqpcqGGOaakiiGacqWFZoWzdaqhaaWcbaGaeeymaeJaeeilaWccbiGae4NAaOgabaGae4xBa0Mae4xDauNae4hDaqNae4xyaeMae4NBa4Mae4hDaqhaaOGaeiilaWIae83SdC2aa0baaSqaaiabbkdaYiabbYcaSiab+PgaQbqaaiab+1gaTjab+vha1jab+rha0jab+fgaHjab+5gaUjab+rha0baakiabcYcaSiabc6caUiabc6caUiabc6caUiabcYcaSiab=n7aNnaaDaaaleaacqGFTbqBcqqGSaalcqGFQbGAaeaacqGFTbqBcqGF1bqDcqGF0baDcqGFHbqycqGFUbGBcqGF0baDaaGccqGGPaqkdaahaaWcbeqaaiabdsha0baaaaa@6AC6@ (*j *= *1, 2, ..., 146*) by Eqs. (10, 11). The EMC vectors for the mutant were employed to simulate the flux distribution for the mutant **v**_*j*_^*mutant *^= (*v*_1*j*_^*mutant*^, *v*_2*j*_^*mutant*^, ..., *v*_7*j*_^*mutant*^)^*t *^(*j *= *1, 2, ..., 146*). Figure 4a shows the spectrums of the estimated flux distributions in the mutant, where the mean vmeanmutant
 MathType@MTEF@5@5@+=feaafiart1ev1aaatCvAUfKttLearuWrP9MDH5MBPbIqV92AaeXatLxBI9gBaebbnrfifHhDYfgasaacH8akY=wiFfYdH8Gipec8Eeeu0xXdbba9frFj0=OqFfea0dXdd9vqai=hGuQ8kuc9pgc9s8qqaq=dirpe0xb9q8qiLsFr0=vr0=vr0dc8meaabaqaciaacaGaaeqabaqabeGadaaakeaacqWH2bGDdaqhaaWcbaGaemyBa0MaemyzauMaemyyaeMaemOBa4gabaGaemyBa0MaemyDauNaemiDaqNaemyyaeMaemOBa4MaemiDaqhaaaaa@3C20@ and the standard deviation are plotted for each flux. The resultant solution spectrums were broad.

First, to explore a representative value for such broad spectrums, we plotted the relative standard deviation of the estimated flux with respect to the mean as shown in Figure 4b. For very small mean values of the estimated fluxes (< 2), the relative standard deviations were large, revealing that much of the flux data were scattered. In contrast, for large values the relative standard deviations were much smaller, indicating that most flux data were located around the mean. These findings suggest that the mean flux with a relatively large value can be representative for the estimated flux. To further confirm if the mean is representative, we plotted the frequency distribution with respect to flux (see Additional file 3). The frequency density is very high around the mean and only a few flux points are scattered away from the mean. Thus, the mean value is regarded as the estimated flux.

Second, to characterize the precision of ECF we compared the mean estimated fluxes with the experimental ones for the *pykF*(-) mutant. The relative model error for each flux (Eq. (15)) is plotted with respect to the experimental flux for the mutant as shown in Figure 4c. As the experimental flux of the mutant increases, the relative model error for flux decreases, showing that ECF simulates high flux values accurately. Out of 30 reactions, ECF estimates 20 fluxes with a low relative model error for flux of less than 0.5.

Third, we provide a physiological validation for the estimated flux distribution. To investigate which EMCs are enhanced in the *pykF*(-) knockout mutant, we calculated the part of Eqs. (7, 8), εi=∏p=1napiβ
 MathType@MTEF@5@5@+=feaafiart1ev1aaatCvAUfKttLearuWrP9MDH5MBPbIqV92AaeXatLxBI9gBaebbnrfifHhDYfgasaacH8akY=wiFfYdH8Gipec8Eeeu0xXdbba9frFj0=OqFfea0dXdd9vqai=hGuQ8kuc9pgc9s8qqaq=dirpe0xb9q8qiLsFr0=vr0=vr0dc8meaabaqaciaacaGaaeqabaqabeGadaaakeaaiiGacqWF1oqzdaWgaaWcbaGaemyAaKgabeaakiabg2da9maarahabaGaemyyae2aa0baaSqaaiabdchaWjabdMgaPbqaaiab=j7aIbaaaeaacqWGWbaCcqGH9aqpcqaIXaqmaeaacqWGUbGBa0Gaey4dIunaaaa@3DB3@, where β is 1 and ε_ι _is 1 for wild-type. The ε_ι _values of the EMs that contain the reactions of 11 (*ppc*) and 12 (*mez*) are greater than 1, whereas the ε_ι _values of the EMs that do not have them are less than 1. These show the fluxes that contain *ppc *and *mez *are enhanced, which are consistent with the fact that the *pykF*-knockout-mediated blockage of the PEP to PYR pathway greatly activates both *ppc *and *mez *enzymes to supply PYR through alternative pathways[11].

#### Effect of the number of integrated enzyme activities

**Figure 5:**
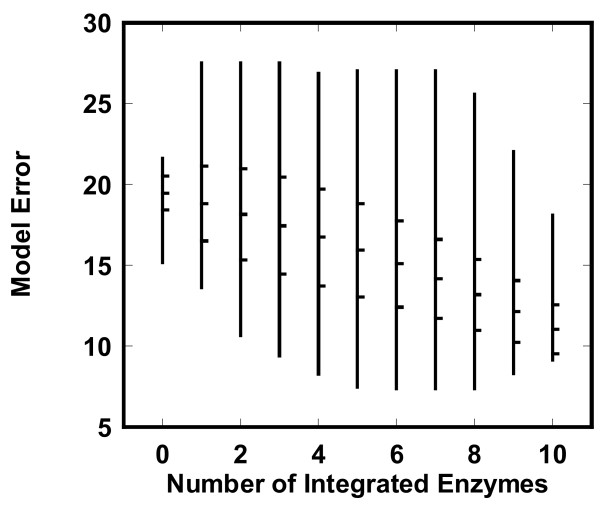
**Effect of the number of integrated enzyme activities on the model error in the *pykF*(-) knockout mutant**. The model error was calculated for all combinations of the enzymes with measured activities. The vertical lines indicate the regions between the minimum and maximum values. Each line has three transverse dashes at the right: The middle dash is the mean of the model error. The upper and lower dashes indicate *m *+ σ and *m *- σ, respectively (*m *and σ are the mean and the standard deviation of the model error, respectively).

To investigate how the number of integrated enzyme activities affects the estimated fluxes in the *pykF*(-) mutant, we calculated the model error profile defined by Eq. (21) for all combinations of the enzymes as shown in Figure 5. The spectrum for the model error is described with respect to the number of integrated enzyme activities. The allowable ranges of the model error were broad, but the mean model error for all the enzyme combinations decreased as the number of integrated enzymes increased. To characterize the relationships between the model error and the number of integrated enzymes, we calculated the standard deviation for each number of integrated enzymes and plotted the frequency distribution with respect to the model error (see Additional file 4). The standard deviations were less than the means and the frequency distribution indicated a high density around the mean. The mean can be regarded as an indicator of the model error profile. The model error decreased with an increase in the number of integrated enzymes, indicating that enzyme data is effectively used by ECF.

We investigated the mechanism of how the allowable model error showed a broad range. We compared the combinations for the incorporated enzymes between the mutants showing underestimated and overestimated model errors, clarifying that the members of the incorporated enzymes and the reaction 7 (*gltA*) are critical. The model error was relatively small when multiple enzymes responsible for branching reactions were incorporated together (data not shown). In contrast, when no enzyme at the branching reactions was integrated, the model error was relatively large. These findings suggest that incorporation of branching reactions can be effective in enhancing estimation accuracy, although it does not change the model error remarkably.

Next, we investigated the relationship between enzyme activities and their associated fluxes. While the flux of the *gltA *reaction in the mutant was almost the same as that in wild-type, the enzyme activity for the mutant was less than a half of that for wild-type. The mutants that incorporate *gltA *show relatively large errors, whereas the mutants without it indicate small errors. Thus, the incorporation of *gltA *is suggested to cause the model error to increase. To confirm it, we excluded the enzyme activity of *gltA *and simulated the model error with respect to the number of integrated enzymes. The allowable ranges of the model error became narrow significantly (data not shown). In the case of *gltA *being integrated, some metabolic factors, such as changes in the concentration of AcCoA, may be involved to compensate the decreased *gltA *activity.

#### Validation of the unique parameter of β

**Figure 6:**
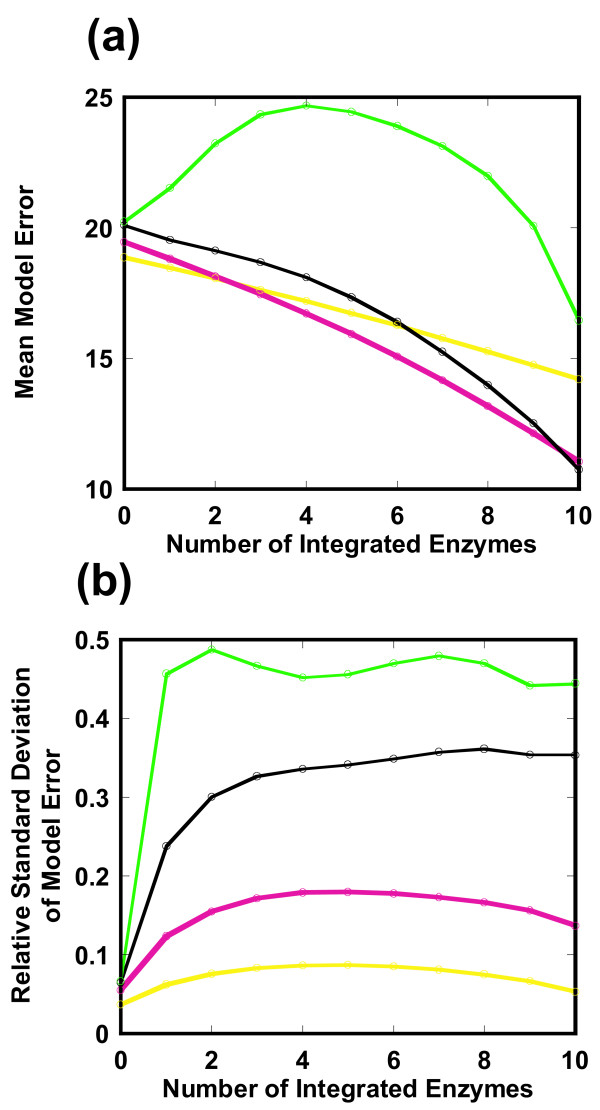
**Optimization of the β factor in the *pykF *(-) knockout mutant**. The model error profile is simulated with respect to the number of integrated enzymes when the β factor is changed as 0.5 (yellow), 1 (red), 2 (black), 4 (green). (a) Mean model error. (b) Relative standard deviation of the model error. The mean for each β is not consistent when the number of incorporated enzymes is zero. This is caused by a non-zero enzyme activity for *pykF *in the mutant.

Here, we demonstrate that a β factor of 1 is a best choice. As shown in Figure 6, we investigated how a change in the β factor affected the mean and relative standard deviation of the model error profile of Eq. (21). The β factor determines the degree by which the change in an enzyme profile affects the flux distribution for a mutant. A β factor of 0 indicates that no enzyme data is used by ECF (Eqs. (7, 8)). In a factor range from 0.5 to 2, the mean model error decreases as the number of incorporated enzymes increases (Figure 6a). At a factor value of 4, the mean model error does not decrease monotonically with respect to the number of incorporated enzymes and the mean model error becomes larger than any other value of β. The mean model error becomes very low at a β factor of 1. In Figure 6b, the relative standard deviation increases as the β factor increases. Since accurate estimation requires that both the mean and the relative standard deviation are small, a β factor of 1 is a reasonable value.

### Generality and applicability of ECF

To guarantee the generality and applicability of ECF, we demonstrate that (i) a β factor of 1 is consistent for available mutants, and (ii) the mean model error decreases with an increase in the number of integrated enzymes, by using *E. coli ppc*[13], *cra*, *fnr *(data not shown), *gnd*[14], *pgi*[15], and *zwf*[14,16] knockout mutants, and an *als*-overexpressing and *pta *(-) knockout mutant of *Bacillus subtilis *(see Additional file 5)[17].

**Figure 7:**
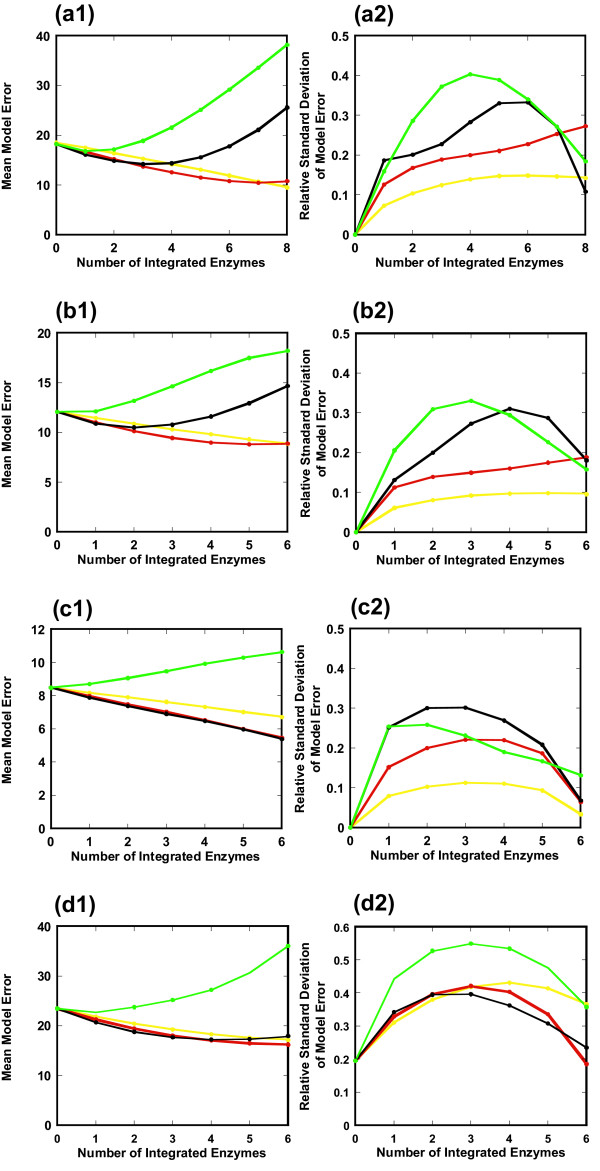
**Applicability and generality of ECF**. Effect of the number of integrated enzymes and the β factor on the model error profile is investigated for various mutants to demonstrate the applicability of the ECF model with a β factor of 1. (a) *ppc*(-) knockout mutant, (b) *cra*(-)knockout mutant, (c) *fnr*(-) knockout mutant in *E. coli*. (d) *als*-overexpressing and *pta *(-) knockout mutant in *B. subtilis*. The mean (a1, b1, c1, d1) and relative standard deviation (a2, b2, c2, d2) of the model error are simulated with respect to the number of integrated enzymes when the β factor is changed as 0.5 (yellow), 1 (red), 2 (black), 4 (green).

**Figure 8:**
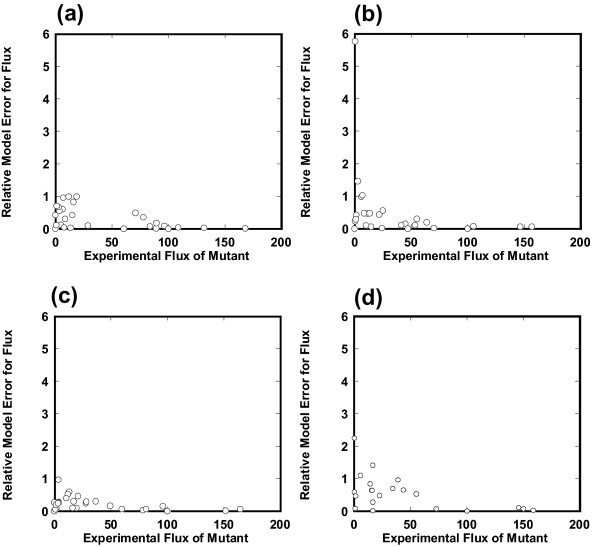
**Consistency of the estimated flux with experimental one for each reaction**. We compared the estimated flux with experimental data for each reaction: (a) *ppc *(-) knockout mutant, (b) *cra *(-) knockout mutant, and (c) *fnr *(-) knockout mutant in *E. coli*. (d) *als*-overexpressing and *pta *(-) knockout mutant of *B. subtilis*. A β factor of 1 is used. The relative model error for flux is plotted with respect to its experimental flux. A small value of the relative model error for flux indicates that the simulated flux is consistent with the experimental one.

As shown in Figure 7, we investigated how a change in the β factor affected the mean and relative standard deviation of the model error of Eq. (21). The model accuracy for the *pgi*, *gnd*, and *zwf *knockout mutants was not significant due to the small number of incorporated enzymes (*n *≤ 4) (data not shown). However, for a relatively large number (*n *≥ 6) of integrated enzymes, the mean model error of the *ppc*, *cra*, and *fnr *knockout mutants and the *als*-overexpressing and *pta *(-) knockout mutant decreased greatly. For these mutants a β factor of 1 is a best choice, which minimizes the mean model error with a relatively small value of the relative standard deviation. Furthermore, the mean model error for a β factor of 1 clearly decreases with an increase in the number of integrated enzymes, indicating that an enzyme activity profile is effectively used by ECF. The comparison between the estimated and experimental fluxes for each reaction is shown in Figure 8, where all available enzyme data are used by ECF and a β factor of 1 is employed. A small value of the relative model error for flux means consistency with experimental data. For a relatively large value of experimental flux, the simulated flux by ECF is rather consistent with the experimental one of these mutants as well as that of the *pykF *knockout mutant (Figure 4c).

## Conclusion

It is important to explore some relationships between an enzyme activity profile and a metabolic flux distribution for rationally designing organism production systems or for understanding the physiological dynamics within a cell. The ECF model is a non-mechanistic model to mathematically link enzyme data to metabolic flux. Assuming that the flux through each EM is synergistically affected by all enzyme activities that belong to the EM, the power-law formula is used to integrate all enzyme activities into EMA. The ECF model successfully links enzyme activities to the flux distributions of *E. coli *and *B. subtilis *mutants, indicating that many enzyme activities affect the flux through each EM simultaneously. In other words, the ECF model is highly applicable to the metabolic models whose flux distribution would be determined by the effects of multiple enzyme activities rather than by a few rate-limiting reactions. In addition, the incorporation of branching reactions is suggested to play a significant role in enhancing model accuracy.

In the power-law formula the β factor determines the degree by which the change in an enzyme profile affects the flux distribution for a mutant. A β factor from 0.5 to 1 is effective in decreasing the model error for all available mutants. A β factor of 1 is a best choice in all experimental mutants, but it does not mean that the change in an elementary mode flux is linear to an enzyme activity because the relationship between a flux and enzymes is complicated than expected from a β factor of 1 as shown in Eqs. (10, 11). A β factor of 1 is not the exact optimal value but an approximate or representative one. The important thing would be to notice that the ECF model decreases the model error at an appropriate value of β rather than to insist biological basis of a β factor of 1.

In the metabolic engineering field, powerful analyses had been proposed to simulate the change in flux distributions in response to environmental or genetic changes. For example, network rigidity had extensively been investigated to reveal how the rigidity of some principal nodes at branch reactions is generated by complex enzyme regulations including allosteric regulation[18]. Such a study reduces the analysis of large-scale metabolic networks to that of the principal nodes, intensively analyzing local kinetics around them to improve flux distributions. The principal nodes showing strong rigidity play a major role in determining the flux distribution in response to different stimulus. On the other hand, the ECF model adopts a different way from such principal node analysis. The ECF model neither considers any allosteric kinetics nor reduces large-scale network analysis to local one. Despite such a plain idea, ECF predicts how the change in enzyme profiles affects the flux distribution. This indicates that the change in an enzyme profile plays a major role in determining flux distributions or it rather reflects the change in the flux distribution.

Finally we show two limitations of ECF. One is that metabolic control analysis (MCA) is not applied to analysis for the ECF model. The other is the limitation of network size. Generally MCA is used to quantify flux control of each reaction by estimating the logarithmic gain of a flux with respect to changes in an enzyme activity [19-21]. In the ECF model, the power factor in Eqs. (7, 8) seems to be a logarithmic gain of the flux through an EM with respect to an enzyme activity. However, MCA may not be applied to the ECF model, because ECF is a non-mechanistic model that neither considers detailed enzyme kinetics, such as allosteric binding of inhibitors and activators, nor the concentrations of substrates. The size of networks analyzed by ECF is limited. Since the number of EMs increases exponentially with the network size, it is hard to analyze large-scale networks with more than hundreds of reactions. A few methods, which divide the network into subsystems by redefining internal and external metabolites [22] or improve the algorithm deriving EMs from a stoichiometric matrix[23], have been proposed to reduce calculation complexity, but they have not fully established yet.

## Methods

### Metabolic model

For more than a decade, the metabolism of *E. coli *has served as a testing ground for network analysis methods. The studies of this metabolic network have been facilitated by the extensive availability of *E. coli *biochemical data. In the present analysis, we simplified the central metabolic networks of glycolysis, the tricarboxylic acid cycle (TCA), and the pentose phosphate pathway (Figure 2 and Table 1), which together contain 16 metabolites and 30 reactions. The strains used were wild-type (*E. coli *K-12) and the knockout mutants (*pykF*, *ppc*, *cra*, *fnr*, *gnd*, *pgi*, and *zwf*), which were constructed by deletion of the corresponding gene from *E. coli *derivative BW25113[24]. In these mutants, the internal metabolic flux had been estimated using results from ^13^C-labeled glucose experiments, and the key enzymes involving the branching reactions (G6P, PYR, AcCoA, OAA, etc) were measured in the main pathway of the TCA cycle and glycolysis [11,13-16].

### Implementation

CADLIVE, which is freely available from , is employed to draw the *E. coli *metabolic network map and describe its corresponding reactions in XML format (SANAC)[25,26]. The generated XML file is converted into a FluxAnalyzer readable format[12]. FluxAnalyzer identified the stoichiometric matrix and elementary modes. The programs for optimization and ECF are written in Matlab (Mathworks Inc., Natick, MA).

## Authors' contributions

HK and KS designed the research. HK, ZQ, and RO performed the simulations and analyzed the results. All authors have read and approved the final manuscript.

## Supplementary Material

Additional file 1 A plain instruction for ECF. Supplementary text. Click here for file

Additional file 2 Supplementary figure 1. Estimation of 73 EMCs in central metabolic pathways of wild type. Click here for file

Additional file 3 Supplementary figure 2. Frequency distributions for the estimated flux in the *pykF*(-) knockout mutant. Click here for file

Additional file 4 Supplementary figure 3. Frequency distributions for the model error in the *pykF*(-) knockout mutant. Click here for file

Additional file 5 Supplementary figure 4 and tables 1 and 2. Data for the *B. subtilis *metabolic network. Click here for file
