# Supplementary material for: Integration of enzyme activities into metabolic flux distributions by elementary mode analysis
Source: BMC Syst Biol. 2007 Jul 18;1:31. doi: 10.1186/1752-0509-1-31 (PMC1973080; doi:10.1186/1752-0509-1-31)
Supplement: Additional file 1 — A plain instruction for ECF. Supplementary text. [file 1752-0509-1-31-S1.pdf]

A plain instruction for ECF

To explain the ECF method intelligibly, it is applied to a plain network model where the EMCs are not uniquely determined. The flux of v_1 is fixed to 100.

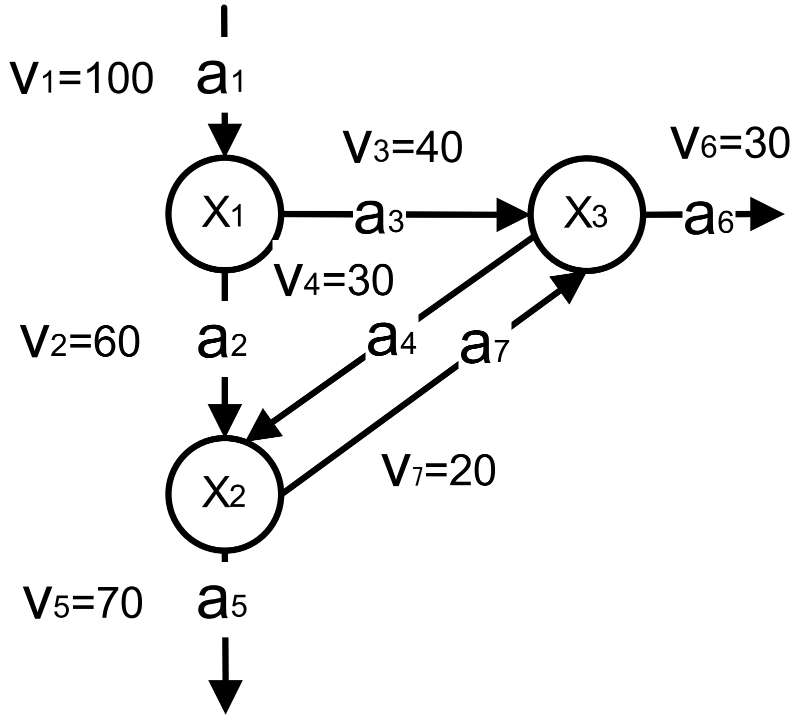

The flux distribution of $\mathbf{v} = (v_1, v_2, ... v_7)^t$ is decomposed into five elementary modes:

$$\begin{pmatrix} v_1 \\ v_2 \\ v_3 \\ v_4 \\ v_5 \\ v_6 \\ v_7 \end{pmatrix} = \lambda_1 \begin{pmatrix} 1 \\ 1 \\ 0 \\ 0 \\ 1 \\ 0 \\ 0 \end{pmatrix} + \lambda_2 \begin{pmatrix} 1 \\ 0 \\ 1 \\ 0 \\ 0 \\ 1 \\ 0 \end{pmatrix} + \lambda_3 \begin{pmatrix} 1 \\ 0 \\ 1 \\ 1 \\ 1 \\ 0 \\ 0 \end{pmatrix} + \lambda_4 \begin{pmatrix} 1 \\ 1 \\ 0 \\ 0 \\ 0 \\ 1 \\ 1 \end{pmatrix} + \lambda_5 \begin{pmatrix} 0 \\ 0 \\ 0 \\ 1 \\ 0 \\ 0 \\ 1 \end{pmatrix}$$

or

$$\begin{pmatrix} 100 \\ 60 \\ 40 \\ 30 \\ 70 \\ 30 \\ 20 \end{pmatrix} = \begin{pmatrix} 1 & 1 & 1 & 1 & 0 \\ 1 & 0 & 0 & 1 & 0 \\ 0 & 1 & 1 & 0 & 0 \\ 0 & 0 & 1 & 0 & 1 \\ 1 & 0 & 1 & 0 & 0 \\ 0 & 1 & 0 & 1 & 0 \\ 0 & 0 & 0 & 1 & 1 \end{pmatrix} \begin{pmatrix} \lambda_1 \\ \lambda_2 \\ \lambda_3 \\ \lambda_4 \\ \lambda_5 \end{pmatrix} = \mathbf{P} \cdot \mathbf{c},$$

where $\mathbf{P}$ is the elementary mode matrix and $\mathbf{c}=(\lambda_1,\lambda_2,..,\lambda_5)^t$ is the EMC vector of wild type. Ten EMC vectors for wild type $(\mathbf{r}_1,\mathbf{r}_2,...,\mathbf{r}_{10})$ are calculated by maximizing or minimizing each λ_i $(i=1,2,..,5)$ as follows:

$$(\mathbf{r}_1,\mathbf{r}_2,...,\mathbf{r}_{10})=\begin{pmatrix} 60 & 40 & 60 & 40 & 40 & 60 & 40 & 60 & 60 & 40 \\ 30 & 10 & 30 & 10 & 10 & 30 & 10 & 30 & 30 & 10 \\ 10 & 30 & 10 & 30 & 30 & 10 & 30 & 10 & 10 & 30 \\ 0 & 20 & 0 & 20 & 20 & 0 & 20 & 0 & 0 & 20 \\ 20 & 0 & 20 & 0 & 0 & 20 & 0 & 20 & 20 & 0 \end{pmatrix}.$$

In a mutant, the activity of a_2 is reduced to a half (0.5) and that of a_4 is 2-fold enhanced. The relative enzyme activity of a mutant to wild type is given by:

$$(\theta_p)=\begin{pmatrix} 1 \\ 0.5 \\ 1 \\ 2 \\ 1 \\ 1 \\ 1 \end{pmatrix}$$

The matrix (α_{pi}) is provided by:

$$(a_{pi})=\begin{pmatrix} 1 & 1 & 1 & 1 & 1 \\ 0.5 & 1 & 1 & 0.5 & 1 \\ 1 & 1 & 1 & 1 & 1 \\ 1 & 1 & 2 & 1 & 2 \\ 1 & 1 & 1 & 1 & 1 \\ 1 & 1 & 1 & 1 & 1 \\ 1 & 1 & 1 & 1 & 1 \end{pmatrix}.$$

For the first and second columns for $(\mathbf{r}_1,\mathbf{r}_2,...,\mathbf{r}_{10})$, the intermediate EMC vectors for the mutant are given by:

$$\mathbf{inter\_r}_1^{mutant}=\begin{pmatrix} 30 \\ 30 \\ 20 \\ 0 \\ 40 \end{pmatrix}=\begin{pmatrix} 60\cdot 0.5^1 \\ 30 \\ 10\cdot 2^1 \\ 0 \\ 20\cdot 2^1 \end{pmatrix} \text{ and } \mathbf{inter\_r}_2^{mutant}=\begin{pmatrix} 20 \\ 10 \\ 60 \\ 10 \\ 0 \end{pmatrix}=\begin{pmatrix} 40\cdot 0.5^1 \\ 10 \\ 30\cdot 2^1 \\ 20\cdot 0.5^1 \\ 0 \end{pmatrix},$$

respectively, where β is set to 1. The others are calculated in the same manner. The resulting intermediate EMCs of $\{\mathbf{inter\_r}_j^{mutant} \mid j=1,2,...,10\}$ are provided by:

$$(\mathbf{inter\_r}_1^{mutant}, \mathbf{inter\_r}_2^{mutant}, ..., \mathbf{inter\_r}_{10}^{mutant}) = \begin{pmatrix} 30 & 20 & 30 & 20 & 20 & 30 & 20 & 30 & 30 & 20 \\ 30 & 10 & 30 & 10 & 10 & 30 & 10 & 30 & 30 & 10 \\ 20 & 60 & 20 & 60 & 60 & 20 & 60 & 20 & 20 & 60 \\ 0 & 10 & 0 & 10 & 10 & 0 & 10 & 0 & 0 & 10 \\ 40 & 0 & 40 & 0 & 0 & 40 & 0 & 40 & 40 & 0 \end{pmatrix}$$

For the vector of each column, the EMC vectors of the mutant $\{\mathbf{r}_j^{mutant} \mid j = 1,2,...,10\}$ are calculated. Here, the first and second columns are used to calculate $\mathbf{r}_1^{mutant}$ and $\mathbf{r}_2^{mutant}$ as follows:

$$\mathbf{r}_1^{mutant} = \begin{pmatrix} 37.5 \\ 37.5 \\ 25 \\ 0 \\ 50 \end{pmatrix} = \frac{100}{(1,0,0,0,0,0,0) \cdot \left(\begin{pmatrix} 1 & 1 & 1 & 1 & 0 \\ 1 & 0 & 0 & 1 & 0 \\ 0 & 1 & 1 & 0 & 0 \\ 0 & 0 & 1 & 0 & 1 \\ 1 & 0 & 1 & 0 & 0 \\ 0 & 1 & 0 & 1 & 0 \\ 0 & 0 & 0 & 1 & 1 \end{pmatrix} \cdot \begin{pmatrix} 30 \\ 30 \\ 20 \\ 0 \\ 40 \end{pmatrix} \right)} \cdot \begin{pmatrix} 30 \\ 30 \\ 20 \\ 0 \\ 40 \end{pmatrix}$$

and

$$\mathbf{r}_2^{mutant} = \begin{pmatrix} 20 \\ 10 \\ 60 \\ 10 \\ 0 \end{pmatrix} = \frac{100}{(1,0,0,0,0,0,0) \cdot \left(\begin{pmatrix} 1 & 1 & 1 & 1 & 0 \\ 1 & 0 & 0 & 1 & 0 \\ 0 & 1 & 1 & 0 & 0 \\ 0 & 0 & 1 & 0 & 1 \\ 1 & 0 & 1 & 0 & 0 \\ 0 & 1 & 0 & 1 & 0 \\ 0 & 0 & 0 & 1 & 1 \end{pmatrix} \cdot \begin{pmatrix} 20 \\ 10 \\ 60 \\ 10 \\ 0 \end{pmatrix} \right)} \cdot \begin{pmatrix} 20 \\ 10 \\ 60 \\ 10 \\ 0 \end{pmatrix},$$

respectively. The flux distributions of the mutant, $\mathbf{v}_1^{mutant}$ and $\mathbf{v}_2^{mutant}$, are calculated as follows:

$$\mathbf{v}_1^{mutant} = \begin{pmatrix} 1 & 1 & 1 & 1 & 0 \\ 1 & 0 & 0 & 1 & 0 \\ 0 & 1 & 1 & 0 & 0 \\ 0 & 0 & 1 & 0 & 1 \\ 1 & 0 & 1 & 0 & 0 \\ 0 & 1 & 0 & 1 & 0 \\ 0 & 0 & 0 & 1 & 1 \end{pmatrix} \begin{pmatrix} 37.5 \\ 37.5 \\ 25 \\ 0 \\ 50 \end{pmatrix} = \begin{pmatrix} 100 \\ 37.5 \\ 62.5 \\ 75 \\ 62.5 \\ 37.5 \\ 50 \end{pmatrix}.$$

and

$$\mathbf{v}_2^{mutant} = \begin{pmatrix} 1 & 1 & 1 & 1 & 0 \\ 1 & 0 & 0 & 1 & 0 \\ 0 & 1 & 1 & 0 & 0 \\ 0 & 0 & 1 & 0 & 1 \\ 1 & 0 & 1 & 0 & 0 \\ 0 & 1 & 0 & 1 & 0 \\ 0 & 0 & 0 & 1 & 1 \end{pmatrix} \begin{pmatrix} 20 \\ 10 \\ 60 \\ 10 \\ 0 \end{pmatrix} = \begin{pmatrix} 100 \\ 30 \\ 70 \\ 60 \\ 80 \\ 20 \\ 10 \end{pmatrix},$$

respectively. The other flux distributions are calculated in the same manner. The mean flux for the mutant is provided by:

$$\mathbf{v}_{mean}^{mutant} = \frac{1}{10}\sum_{j=1}^{10} \mathbf{v}_j^{mutant} = \begin{pmatrix} 100 \\ 33.75 \\ 66.25 \\ 67.5 \\ 71.25 \\ 28.75 \\ 30 \end{pmatrix}.$$
