# Supplementary material for: Integration of enzyme activities into metabolic flux distributions by elementary mode analysis
Source: BMC Syst Biol. 2007 Jul 18;1:31. doi: 10.1186/1752-0509-1-31 (PMC1973080; doi:10.1186/1752-0509-1-31)
Supplement: Additional file 2 — Supplementary figure 1. Estimation of 73 EMCs in central metabolic pathways of wild type. [file 1752-0509-1-31-S2.pdf]

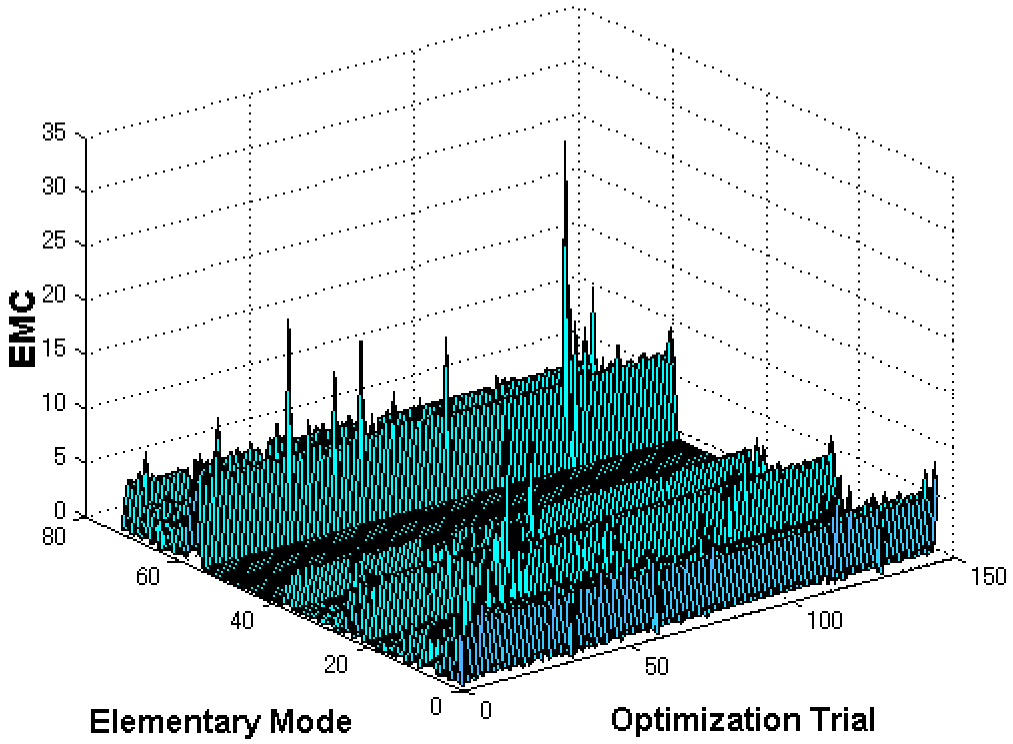

Supplementary Figure 1 Estimation of 73 EMCs in central metabolic pathways of wild type
The EMCs with respect to the (*2j-1* and *2j*)-th optimization trial are calculated by maximizing or minimizing the *j*-th EMC (*j=1,2,...,73*).
