# Supplementary material for: Integration of enzyme activities into metabolic flux distributions by elementary mode analysis
Source: BMC Syst Biol. 2007 Jul 18;1:31. doi: 10.1186/1752-0509-1-31 (PMC1973080; doi:10.1186/1752-0509-1-31)
Supplement: Additional file 4 — Supplementary figure 3. Frequency distributions for the model error in the pykF(-) knockout mutant. [file 1752-0509-1-31-S4.pdf]

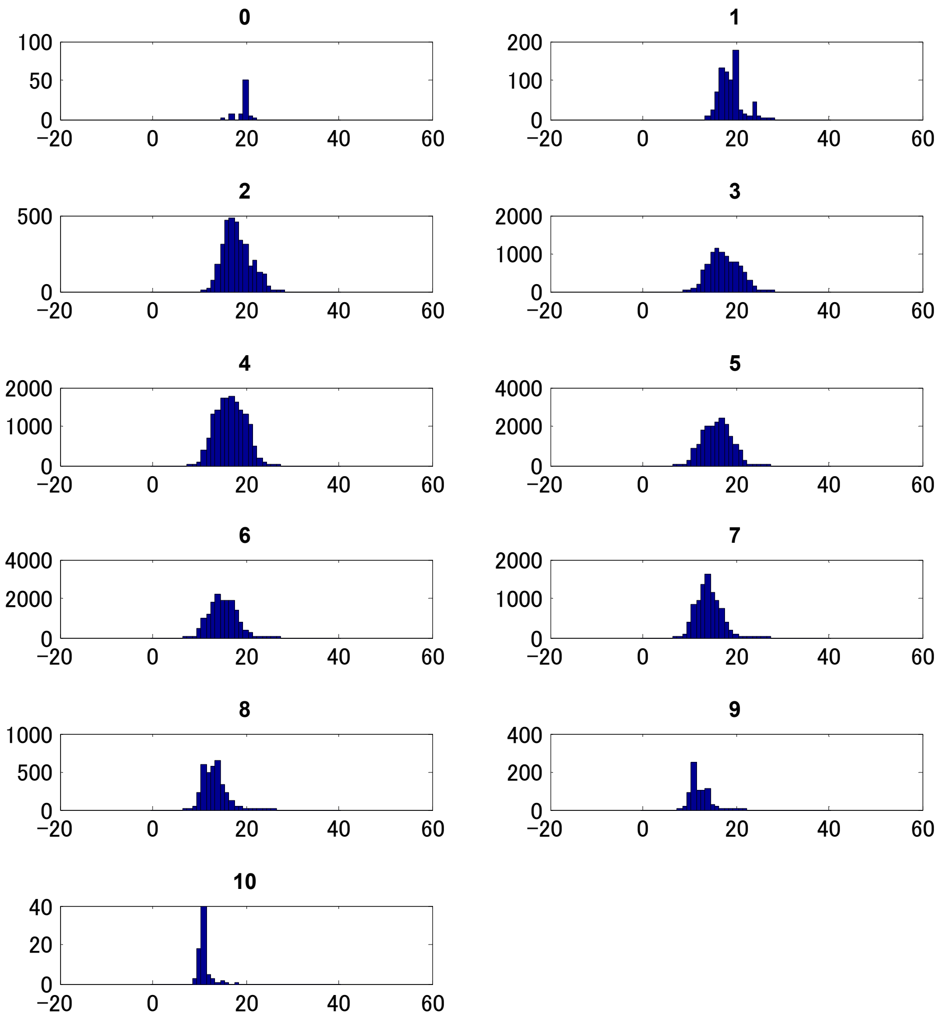

Supplementary Figure 3 Frequency distributions for the model error in the *pykF(-)* knockout mutant

The number of integrated enzymes is described above each figure. The horizontal axis indicates the model error and the vertical axis is the frequency.
