# Supplementary material for: Integration of enzyme activities into metabolic flux distributions by elementary mode analysis
Source: BMC Syst Biol. 2007 Jul 18;1:31. doi: 10.1186/1752-0509-1-31 (PMC1973080; doi:10.1186/1752-0509-1-31)
Supplement: Additional file 5 — Supplementary figure 4 and tables 1 and 2. Data for the B. subtilis metabolic network. [file 1752-0509-1-31-S5.pdf]

Data for the *B. subtilis* metabolic network

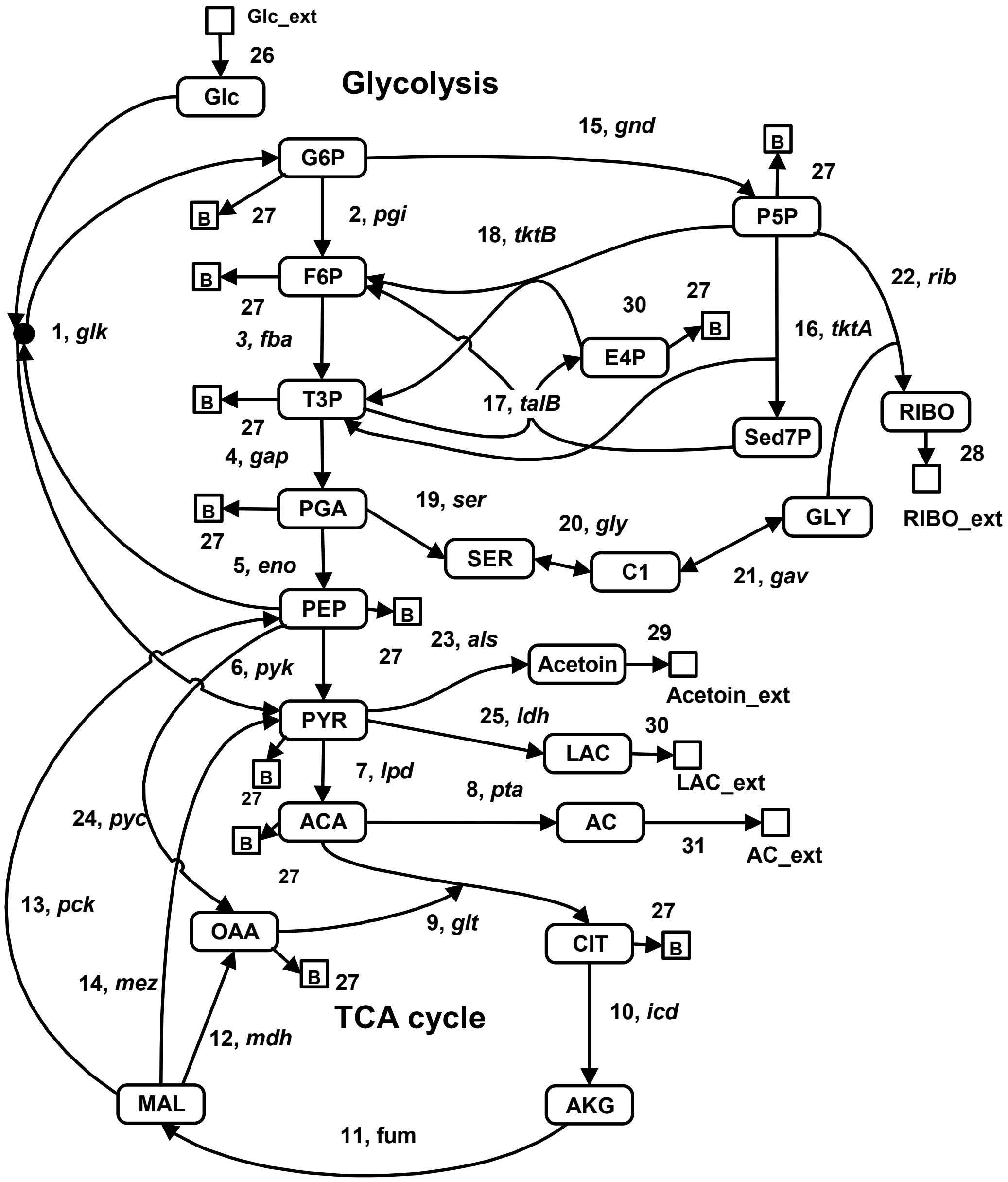

Supplementary Figure 4 *Bacillus subtilis* central metabolic network

The numbers in the figure are those of reactions shown in **Supplementary Table 1**. The gene name is representative of the genes related to the reaction. The metabolites indicated by squares, e.g., B (biomass), Glc_ext (environmental glucose) and Ac_ext (environmental acetate), are external. All other metabolites, enclosed by rounded rectangles, are internal.

B. subtilis is an important model microorganism in the field of metabolic engineering for the production of riboflavin or acetoin [1]. A typical biochemical network was reconstructed where there are 30 metabolites and 31 reactions for *B. subtilis* grown on glucose. The *pta*(-) knockout and acetolactate synthase (*als*) overexpressing mutant of *B. subtilis* RH33::[pRB63] was denoted as *B. subtilis* RH36 which was constructed from parental strain *B. subtilis* RH33 [2]. The flux distributions and enzyme activities of wild type (*B. subtilis* RH33) and mutant (*B. subtilis* RH36) were presented (**Supplementary Table 2**).

1. Sauer U, Cameron DC, Bailey JE: **Metabolic capacity of *Bacillus subtilis* for the production of purine nucleosides, riboflavin, and folic acid**. *Biotechnol Bioeng* 1998, **59**(2):227-238.
2. Zhu Y, Chen X, Chen T, Zhao X: **Enhancement of riboflavin production by overexpression of acetolactate synthase in a *pta* mutant of *Bacillus subtilis***. *FEMS Microbiol Lett* 2007, **266**(2):224-230.

Supplementary Table 1 Reactions from the *B. subtilis* central metabolic network for the production of riboflavin and acetoin

Abbreviation: GLC Glucose; G6P Glucose-6-phosphate; F6P Fructose-6-phosphate;T3P Glyceraldehyde-3-phosphate; PGA 3-phosphoglyceric acid; PEP Phosphoenol pyruvate; SED7P Seduheptulose-7-phosphate; E4P Erythrose-4-phosphate; RIBO Riboflavin; GLY Glycine; SER Serine; PYR Pyruvate; ACA Acetyl coenzyme; AC Acetate;Cit Citrate; OAA Oxaloacetate ; MAL Malate; FUM Fumarate ; C1 Methyl group bound to tetrahydrofolate ; P5P Pentose-5-phosphate; LAC Lactate; Glc_xt External glucose; RIBO_ext Riboflavin external; Acetoin_ext Acetoin external; LAC_ext Lactate external; AC_ext Acetate external.

Reaction	Gene name	Enzyme	Chemical reaction
1	*glk*	Glucokinase	GLC => G6P
2	*pgi*	Phosphoglucoisomerase	G6P <==> F6P
3	*pfkA, pfkB*<br>*fba*<br>*tpi*	Phosphofructokinase<br>Fructose-16-bisphosphatate aldolase<br>Triphosphate isomerase	F6P => 2 T3P
4	*gapA*<br>*pgk*<br>*gpmA*	Glycelaldehyde-3-phosphate dehydrogenase<br>Phosphoglycerate kinase<br>Phosphoglycerate mutase I	T3P <==> PGA
5	*eno*	Enolase	PGA <==> PEP
6	*pyk*	Pyruvate kinase	PEP => PYR
7	*lpd*	Pyruvate dehydrogenase	PYR => ACA + CO2
8	*pta, ackA*	Acetyl-CoA synthetase	ACA => AC
9	*gltA*	Citrate synthase	OAA + ACA => CIT + CO2
10	*icdA*<br>*suc, lpd*<br>*sdhC*	Isocitrate dehydrogenase<br>2-ketoglutarate dehydrogenase<br>Succinyl-CoA synthetase<br>Succinate dehydrogenase	CIT => FUM + CO2
11	*fum*	Fumarate hydratase	FUM <==> MAL
12	*mdh*	Malate dehydrogenase	MAL <==> OAA
13	*pck*	Phosphoenolpyruvate carboxykinase	OAA => PEP + CO2
14	*mae*	Malic enzyme	MAL => PYR + CO2
15	*gnd*	6-phosphoglycononate dehydrogenase	G6P => P5P + CO2
16	*rpi*<br>*rpe*<br>*tptA*	Ribose-5-phosphate isomerase A<br>Ribose phosphate 3-epimerase<br>Transketolase I	2 P5P <==> S7P + T3P
17	*talB*	Transaldolase B	S7P + T3P <==> F6P + E4P
18	*tktB*	Transketolase II	P5P + E4P <==> F6P + T3P
19	*ser*	3-phosphoglyceric acid dehydrogenase	PGA => SER
20	*gly*	Serine hydroxymethyltransferase	SER <==> GLY + C1
21	*gcv*	Glycine synthase	C1 + CO2 <=> GLY
22	*rib*	Riboflavin kinase	3 P5P + GLY => RIBO
23	*als*	Acetolactate synthase	2 PYR => Acetoin
24	*pyc*	Pyruvate carboxylase	PYR + CO_2 => OAA
25	*ldh*	Lactate dehydrogenase	PYR => LAC
26	-	Glucose uptake	Glu_ext => GLC
27	-	Materials are used for biomass synthesis.	=> Biomass
28	-	Membrane transport reaction	RIBO => RIBO_ext
29	-	Membrane transport reaction	Acetoin => Acetoin_ext
30	-	Membrane transport reaction	LAC => LAC_ext
31	-	Membrane transport reaction	AC => AC_ext

Supplementary Table 2 Experimental data of flux and enzyme activities in wild type and the mutant in *B. subtilis*

The glucose uptake flux was normalized to 100. The relative enzyme activities in the mutant were normalized by those in the wild type. Enzymes were extracted from the cultured cells and their activities were measured *in vitro*.

	Flux in the wild type	Flux in the mutant	Relative enzyme activities in the mutant
1	100.0	100.0	1
2	40.8	43.8	1
3	71.7	72.9	1
4	157.3	158.9	1
5	148.0	149.9	1
6	143.9	146.0	1
7	108.0	34.5	1
8	83.7	0.0	0.0001
9	11.9	22.7	3.3
10	5.7	16.8	2.3
11	5.7	16.8	1
12	5.7	16.7	1
13	0.0	0.0	1
14	0.0	0.1	1
15	58.3	55.3	0.8
16	16.8	15.9	1
17	16.9	15.9	1
18	15.1	14.2	1
19	1.3	1.2	1
20	1.3	1.2	1
21	0.4	0.4	1
22	1.6	1.6	1
23	0.6	39.1	4.9
24	17.3	16.7	1
25	0.4	0.4	0.8
26	100.0	100.0	1
27	5.8	5.5	1
28	1.6	1.6	1
29	0.6	39.1	1
30	0.4	0.4	1
31	83.7	0.0	1
